# A two-step site and mRNA-level model for predicting microRNA targets

**DOI:** 10.1186/1471-2105-11-612

**Published:** 2010-12-31

**Authors:** Takaya Saito, Pål Sætrom

**Affiliations:** 1 Department of Cancer Research and Molecular Medicine, Norwegian University of Science and Technology, NO-7489 Trondheim, Norway; 2 Department of Computer and Information Science, Norwegian University of Science and Technology, NO-7491 Trondheim, Norway

## Abstract

**Background:**

Despite experiments showing that the number of microRNA (miRNA) target sites is critical for miRNA targeting, most existing methods focus on identifying individual miRNA target sites and do not model contributions of multiple target sites to miRNA regulation. To address this possible fault, we developed a miRNA target prediction model that recognizes the individual characteristics of functional binding sites and the global characteristics of miRNA-targeted mRNAs.

**Results:**

Benchmark experiments showed that this two-step model generally had a higher overall performance than other established miRNA target prediction algorithms and that the model was especially suited to identify true miRNA targets among genes that all contain conserved target sites.

**Conclusions:**

This improved performance could partly be explained by the model not relying on conservation when predicting targets. The critical factors for the model's performance, however, were mRNA-level features that characterized the number and strength of individual target sites within the mRNA. The model is available for online predictions or as pre-computed predictions on the human genome http://tare.medisin.ntnu.no/mirna_target.

## Background

**Figure 1:**
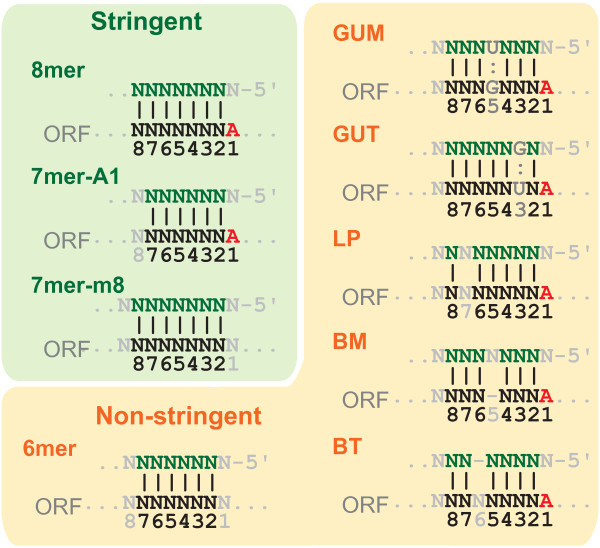
**miRNA seed types**. Nine seed types are categorized in two groups; "Stringent" (8mer, 7mer-A1, and 7mer-m8) and "Non-stringent" (6mer, GUM, GUT, LP, BM and BT). GUM and GUT allow one GU wobble in the seed region. GUM has the U on miRNA whereas GUT has the U on the target. LP, BM and BT allow one mismatch. LP has one loop, BM has one bulge on miRNA, and BT has one bulge on the target.

MicroRNAs (miRNAs) are a class of non-coding RNAs that can regulate many protein coding genes by base-paring to messenger RNA (mRNA) targets [1]. Their roles in gene regulation have been identified in numerous biological processes, such as developmental timing, apoptosis, and cell proliferation [2,3]. The precise mechanism of miRNA targeting is unknown, but animal miRNAs have a small region called "seed" site (Figure 1), which is located at positions 2-7 of the 5' end of miRNAs and is known to contribute to target recognition significantly [4]. Most target sites are found in the mRNA 3' untranslated region (UTR) [1,5,6] and are well conserved among closely related species [2]. A high number of coding genes, except for those with short 3' UTRs such as house keeping genes, are likely regulated by one or multiple miRNAs [7].

Because of the important genome-wide regulatory roles of miRNAs, many computational approaches have been developed to obtain high-throughput genome-wide miRNA target predictions in animals [4,8,9]. Most existing algorithms first perform sequence search on 3' UTRs to find regions that have complementarity to miRNAs preferably at their seed sites. As this initial step usually results in thousands of potential target sites and many false positives, most algorithms take additional features into consideration; for example, evolutionary conservation filters can decrease the false positive rate, but such filters are effective only for conserved miRNAs with sites of conserved function. Several other features have been experimentally and computationally identified, and we have categorized them into two groups: (i) individual target site level features and (ii) global mRNA level features. The six target site level features currently known are (i.a) site conservation, (i.b) additional base paring in 3' portion of miRNA, especially at positions 13-16 of miRNA [5,10], (i.c) AU-rich context within 30 nucleotides upstream and downstream from the seed site [5], (i.d) avoidance of the region from the stop codon to 15 nt downstream in 3' UTR [5,11], (i.e) tendency of targeting near both ends of 3' UTR when the length of the 3' UTR is > 2000 [5,12], and (i.f) site accessibility [13,14]. Furthermore, there are three known mRNA level features: (ii.a) high cooperativity of two miRNA target sites when the distance between them is 17-35 nt [15], (ii.b) length of 3' UTR [4], and (ii.c) the number of potential target sites per 3' UTR [16].

Although some algorithms base their predictions on only one of these additional features, such as conservation [12] or accessibility [13,14], the algorithms commonly use different combinations of features [9]. Most existing algorithms focus on identifying individual target sites, however; few model miRNA targeting at the level of the mRNA and only PicTar tries to explicitly model the contribution of multiple target sites to miRNA regulation [17].

Here, we describe a novel two-step classification model that recognizes the individual characteristics of functional binding sites and the global properties of mRNAs regulated by candidate miRNAs. We base the model on support vector machines (SVM) [18], use large-scale microarray datasets to train and test the model, and benchmark the model against five popular algorithms - TargetScan [5], miRanda (MicroCosm) [16,19], PicTar, PITA [13], and mirTarget2 [20]. As these five algorithms represent different prediction strategies and can have different strengths and weaknesses, we have used six different benchmarks that account for overall predictions, highly specific predictions, predictions of conserved sites, and method-specific data variations. Our method performs better than or equivalent to the other existing algorithms in the six benchmarks when tested on different cross-validation experiments or on an independent test set.

## Results and Discussion

Our goal was to create and test a miRNA target prediction approach that modeled both the characteristics of individual target sites and the global properties of mRNAs regulated by candidate miRNAs. Although there are few experimentally validated miRNA targets, several datasets from microarray experiments where miRNAs were ectopically expressed are available for public use [5,6,21]. High-throughput proteomics experiments have shown that most miRNA targets are regulated at both the mRNA and protein level [22,23], which means that these microarray data are relevant and useful for developing miRNA targeting models. In addition, there are several microarray experiments for small interfering RNA (siRNA) off-target effects [24,25]. Transfected siRNAs are known to act like miRNAs [26,27] and degrade numerous unintended (off-target) mRNAs. Consequently, microarray datasets for siRNA off-targets can be used to reveal aspects of miRNA regulation. Unlike miRNA target sites, however, off-target sites for such artificial and exogenous siRNAs are not evolutionary conserved.

We collected four such microarray datasets - two miRNA and two siRNA datasets - to use as training data to develop our method. Based on an analysis of how different seed types covered the positive and negative target candidates within these datasets (see Additional file 1:Supplementary Results), we chose to develop a target-site model that included both stringent and non-stringent seed sites. Our hypothesis was that by including non-stringent seed sites and training two separate SVMs on target site and mRNA-level features, we would create an accurate miRNA target prediction method with high sensitivity and overall prediction performance.

### SVM prediction performs well with target site features

The first step of our two-step SVM classification approach was to construct a target site level classifier that can separate positive target sites from negative target sites. To construct this target site level classifier, we included several features known or presumed predictive of miRNA targeting (Additional file 1:Table S1). Four sub-steps were applied and then iterated until the most effective classifier was found. First, 10-fold cross-validation was performed to evaluate the prediction ability. Second, a receiver operating characteristic (ROC) curve was plotted to visualize the result of the 10-fold cross-validation and the area under the ROC curve (AUC) was used as a performance measure. The ROC curve and its AUC value, or "ROC score", is a simple but powerful measure of overall classification performance as the curve shows a classifier's sensitivity and specificity across all thresholds. Third, as an SVM can take both linear and non-linear kernels with different parameters, models with different kernels were assessed. Fourth, to check the classifier's robustness across different experimental settings, classifiers were constructed from only three microarray experiments instead of four and tested on the remaining microarray set.

**Figure 2:**
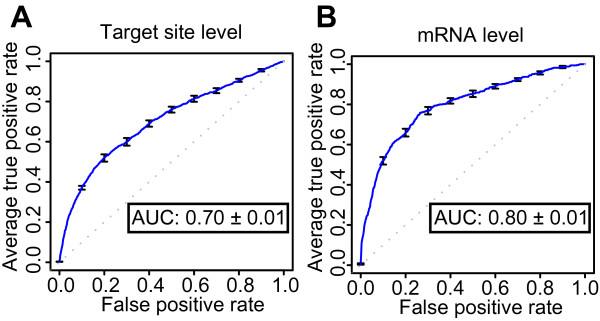
**Target site and mRNA level classifiers provide robust and accurate predictions**. The ROC curve shows the average true positive rate (sensitivity) vs. false positive rate of the10-fold cross-validations for the target site (A) and mRNA level (B) classifiers. Error bars show standard errors; the dotted line illustrates random prediction; AUC indicates the average and the standard error of the average of the area under the curve (AUC; ROC Score) of the ten individual cross-validation test results

**Figure 3:**
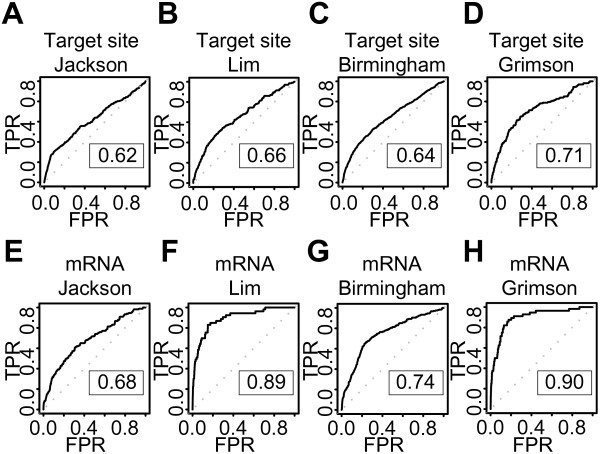
**Target site and mRNA level classifiers generalize to microarray datasets not seen during training**. The ROC graphs show the performance of four target site level classifiers (A-D) and four mRNA level classifiers (E-H) trained on three microarray datasets and tested on the remaining one dataset. Dotted lines illustrate random prediction. The ROC score is shown in the box. TPR indicates True positive rate, while FPR indicates False positive rate. Figure headings state the dataset used for testing.

These iterative tests found that a classifier with non-linear, homogeneous polynomial kernel (parameters degree 5 and cost factor 2) showed the best performance with the ROC score 0.70 (Figure 2A). The small standard errors in the ROC plot indicated that all classifiers from this 10-fold cross-validation had similar prediction power and that the models gave robust classifications. This conclusion was supported by similar results for models trained on three of the four microarray experiments (Additional file 1:Figure S1). Moreover, all the four classifiers from different combinations of three microarray datasets could classify the remaining one dataset with good ROC scores (Figure 3A-D). These results indicated that our target site level classifiers were stable and accurate across experimental conditions, irrespective of whether the sequences were miRNAs or siRNAs.

### Seed type shows the strongest influence on target site level training

It is important to identify the influence of the features on the SVM's prediction accuracy, as this can reveal new information about miRNA target recognition. To evaluate the influence of all the target site level features used in our model, 10-fold cross-validation was performed repeatedly with eliminating only one feature at a time, and then ROC scores were compared. These experiments showed that the "seed type" was the strongest feature with a reduced ROC score of 0.03 (Additional file 1:Table S2). Reduced ROC scores indicate that the feature is important, as the SVM would have more prediction power with the feature included. Other features showed little or no decline of ROC scores, but none had substantial negative effects.

### Genes with long 3' UTRs tend to have few positive target sites in microarray experiments

**Figure 4:**
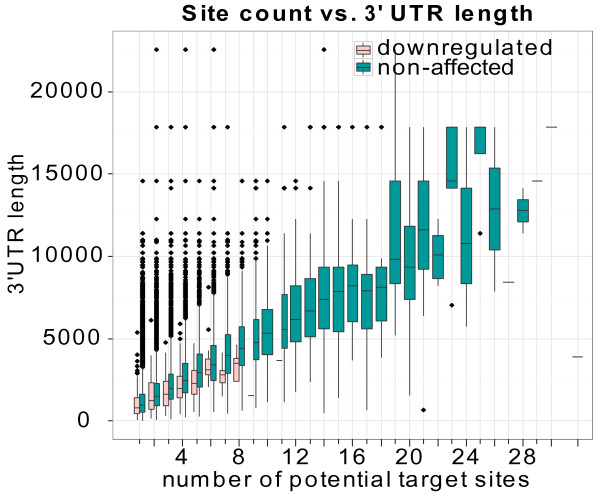
**Fewer positive target sites for genes with long 3' UTRs**. The boxplot shows the lengths of 3' UTRs grouped by the number of potential target sites in the training dataset. Red boxes represent down regulated UTRs, whereas blue boxes represent unaffected UTRs. No down-regulated genes have more than 8 potential target sites and few down-regulated genes have long 3' UTRs (> 5000 nt). The range of potential target site number is from 1 to 28 plus 32 and 38.

While constructing and analyzing the mRNA level training data, we noticed that genes with more than 8 potential target sites appeared to be underrepresented among the positives. This was contrary to our expectations, as experimental data indicate that additional target sites give increased target repression [28]. Additional analyses revealed two trends. First, genes with long 3' UTRs (> 5000 nt) did generally not appear to be miRNA targets - irrespective of the genes' number of potential miRNA target sites (Figure 4). Second, positive targets generally had a higher density of potential target sites (Kolmogorov-Smirnov test, p-value: 4e-11) - that is, number of sites divided by the 3' UTR length - than negative target candidates had (Additional file 1:Figure S2).

One possible explanation for this result is competition between transfected RNAs and endogenous miRNAs [29]. Genes with long 3' UTRs tend to be targeted by several endogenous miRNAs, therefore the transfected RNAs may have less effect on these genes. Another possible explanation for this result is that genes with long 3' UTRs have fewer target sites within these active regions because active sites are preferentially located close to the stop codon or poly-A site within long 3' UTRs [5]. However, we found that the positive targets and negative target candidates had a similar number of target sites within the regions close to the stop codon and poly-A site (data not shown). Thus, a high density of target sites within the middle region of long 3' UTRs appeared to be important for target regulation.

We used two approaches to take this unexpected distribution of true positive targets into consideration. First, a new binary feature was introduced to distinguish the genes with less than 7 potential target sites from the others. Second, 1000 randomly selected non-target genes with more than 7 target sites were explicitly added to the training data. Because of a practical limitation on the maximum number of training data, most of these none-target genes with long 3' UTRs would otherwise have been excluded if we had constructed the training data by random sampling. These two approaches had a positive effect on target predictions for genes with many target site candidates (Additional file 1:Figure S3).

### mRNA features improve SVM predictions

The second step of our two-step SVM classification approach was to construct an mRNA level classifier that can separate down-regulated genes from non down-regulated genes. This subdivision enabled us to incorporate features related to the predicted strength of individual target sites and the distance between these sites that had not been used in other algorithms before (Additional file 1:Table S3). We used the same four steps as for the target site level to find the most effective mRNA level classifier.

The classifier showed the best performance when a linear kernel with default parameters was used (ROC score 0.80). As for the target site level, the ROC curve for the classifier had small standard errors, indicating robust classifications (Figure 2B). Target predictions also retained good performance with classifiers trained with only three microarray datasets (Additional file 1:Figure S4). These classifiers could also predict the remaining data set with good accuracy (Figure 3E-H), but the classifiers showed increased variation between the datasets compared with the corresponding target site level classifiers. Whereas the mRNA level classifiers greatly improved upon the target site level classifiers' performance on the miRNA data (compare panels B and F, and D and H in Figure 3), the mRNA level classifiers gave smaller improvements on the siRNA data (panels A and E, and C and G in Figure 3). Thus, the targets for exogenous siRNAs were more difficult to predict than the targets for endogenous miRNAs were - at least in the datasets used in these experiments. This could not be explained by different preferences for strand loading between the miRNAs and siRNAs, as removing the siRNAs where the intended guide strand was not clearly preferred for RISC incorporation did not improve the SVM's performance (data not shown). Despite these differences, the mRNA level classifier showed good improvement compared with the target site level classifier and could predict target genes with high accuracy.

### Number and strength of putative miRNA target sites strongly predict miRNA regulation

As for the target site level features, we wanted to determine to what extent the different mRNA level features influenced target predictions. We therefore performed a similar evaluation of feature influence for the mRNA level. The result showed that the "distribution of discriminant values" was the strongest feature with a reduced ROC score of 0.04 (Additional file 1:Table S4). Eliminating other features had little effect on the ROC scores, but additional analyses showed that at least the target-site distance features contributed to separate the down-regulated from the unaffected genes (see Additional file 1:Supplementary Results). As the "distribution" feature essentially counted the number of high-scoring putative target sites within the mRNA, these results showed that strong target sites are important for miRNA regulation.

The target site level feature analyses indicated that target site accessibility and conservation had little or no effect on the SVM's predictive performance and additional analyses at the mRNA level confirmed these results (see Additional file 1:Supplementary Results). As computing site accessibility and conservation require substantial computational resources, we excluded these features from the final model. Recently, a tool based on support vector regression (SVR) reported improved target prediction performance [30]. We also tested whether using SVR instead of classification would further improve the results, but instead found that SVR gave reduced performance (see Additional file 1:Supplementary Results).

### Two-step SVM shows strong prediction ability and outperforms other algorithms when tested on independent dataset

Both the 10-fold cross-validation and single dataset hold-out experiments showed that the two-step SVM classifiers could predict miRNA target sites in unseen data with high accuracy. Nevertheless, to further test the SVM classifiers, we evaluated the classifiers on an independent test set and compared their performance with those of other existing target prediction algorithms. We included seven popular miRNA target prediction algorithms - PITA All, PITA Top, TargetScan, TargetScan with conserved genes, miRanda (MicroCosm), mirTarget2, and PicTar - in the comparisons, and used the Linsley dataset because only mirTarget2 had used this microarray dataset as a training set. The predictions of three algorithms - our SVM approach, PITA All, and TargetScan - were generated without conservation information, but the predictions of the other algorithms were generated with conservation information as features or filters. We included mirTarget2 as a reference - despite mirTarget2 using the Linsley dataset for training - because the algorithm, similar to our two-step SVM, adopted a machine learning approach.

The algorithms' predictions had little overlap because all algorithms used different definitions of potential target sites. Hence, it was important to use different datasets to assess and compare the algorithms' prediction performance precisely. We used six types of datasets as benchmarks; these were, ROC with All genes, ROC_10*n_, ROC with 7mer + Conservation, and ROC with TargetScan, miRanda, and PicTar datasets (see Additional file 1:Supplementary Methods).

**Figure 5:**
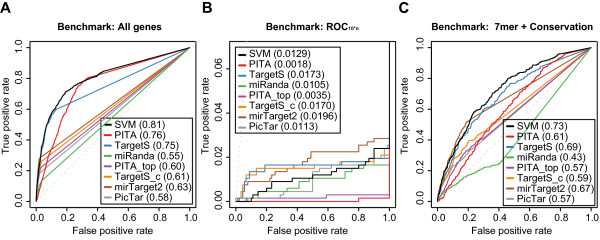
**Benchmarks show the performances of 8 different prediction algorithms**. Receiver operating characteristic (ROC) graphs show the performances of 8 different target prediction algorithms - SVM, PITA All (PITA), PITA Top (PITA_top), TargetScan (TargetS), TargetScan with conserved genes (TargetS_c), MicroCosm miRanda (miRanda), mirTarget2 and PicTar - on the Linsley dataset. Dotted lines illustrate random prediction. The ROC scores are shown in the legend box. The benchmarks used for the evaluation were (A) ROC with All genes, (B) ROC10*n, and (C) ROC with 7mer + Conservation

The "ROC with All genes" dataset was comprised of all the records from the microarray dataset. Down regulated (positive) and unaffected (negative) genes were decided solely by the microarray results regardless of seed matching or any other definitions set by different algorithms. The purpose of using this dataset was to evaluate the algorithms' overall performance on the entire microarray experiment. The resulting ROC curves showed that our SVM approach outperformed the other seven algorithms in terms of sensitivity and overall target recognition (Figure 5A). The number of positive genes found by our SVM approach was 564, which was the largest among all the algorithms (Additional file 1:Table S5). In comparison, PITA All found a similar number of positive genes, but its predictions were less specific than our SVM's predictions were. At similar true positive rates, PITA All returned more false positives, which resulted in a difference in ROC score of 0.05 between SVM and PITA All. Similar to PITA, miRanda returned less specific predictions than the other algorithms did, although miRanda's predictions were more specific than PITA All's at similar true positive rates. miRanda also had the lowest sensitivity of the different algorithms. Apart from differences in sensitivity, the remaining algorithms had similar prediction specificities; mirTarget2 appeared to give slightly more specific predictions than the other algorithms did, but our SVM appeared to have the highest specificity of the methods for which the Linsley set truly was an independent test set. These differences were small, however, and only apparent for the algorithms' most sensitive predictions.

To elucidate the algorithms' performance at highly specific target predictions, we turned to the "ROC_10*n_" benchmark (Figure 5B), which is a modified version of ROC_50 _[31] that shows the algorithms' performance up to 10 * n false positive predictions (n is the number of miRNAs in the dataset; n = 9 for the Linsley set). Although the SVM's ROC_10*n _score is slightly lower than those of the two TargetScan versions and mirTarget2, the SVM again has the highest sensitivity of the algorithms (excluding mirTarget2 and PicTar). Similarly, PITA All again shows very low sensitivity, whereas PicTar and miRanda have similar performance. Thus, the results from the ROC_10*n _benchmark indicate that our SVM approach has very good performance with top scored genes (Figure 5B).

As many of the algorithms only considered stringent seed types and filtered predictions based on conservation, we constructed the "ROC with 7mer + Conservation" benchmark. The benchmark only consisted of the genes with stringent seeds found in conserved regions. Consequently, the benchmark essentially only considered the most likely candidate miRNA target genes and showed how good the different algorithms are at finding the real targets among genes that all are likely targets. Despite that our approach was not optimized for this type of dataset, the SVM delivered more sensitive and specific predictions than the other methods (Figure 5C; again excluding mirTarget2). Interestingly, the algorithms optimized for recognizing conserved stringent seed targets - TargetScan with conserved genes, PITA Top and PicTar - all had markedly lower performance than the SVM. One likely explanation for this result is that other non-conserved seeds within the genes are important for miRNA targeting as well. Supporting this hypothesis, TargetScan - which also considers non-conserved seeds - had a markedly better performance than TargetScan with conserved genes. miRanda showed especially poor performance on the conservation benchmark, but this was likely because the miRanda predictions had very few overlaps with the benchmark dataset due to different conservation filtering. Indeed, miRanda had better performance on its method-specific benchmarks (Figure S5, S7, and S9). The three method-specific benchmarks that use different definitions of sequence conservation and separate benchmark tests on the four training datasets also showed similar results as the 7mer + Conservation benchmark (Additional file 1:Figure S5-S9). Thus, lack of conservation does not guarantee lack of function, and effectively incorporating all seed types in a common prediction framework, such as our SVM, appears to be essential for correctly prioritizing lists of candidate miRNA targets.

### Two-step SVM's improved performance holds when detecting protein level targets

**Figure 6:**
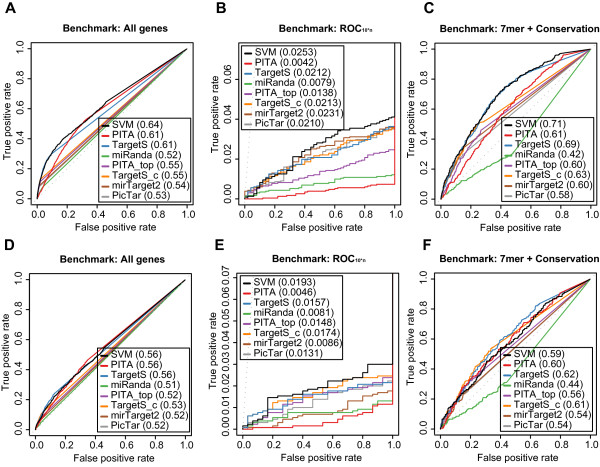
**Benchmark results on proteomics data confirms the two-step SVM's strong performance**. Receiver operating characteristic (ROC) graphs show the performance of 8 different target prediction algorithms on the two proteomics datasets, Selbach (A, B, and C) and Baek (D, E, and F). The benchmarks used for the evaluation were (A) and (D) ROC with All genes, (B) and (E) ROC10*n, (C) and (F) ROC with 7mer + Conservation. See Figure 5 for details regarding the algorithms.

Although several recent studies have suggested the possibility of mRNA repression as miRNA's major regulatory mode [22,23], it is still interesting to verify the prediction power at both mRNA and protein levels. We therefore used two publically available proteomics datasets of miRNA targeting, Baek [22] and Selbach [23], as training sets to create a new proteomics-based two-step SVM classifier and as independent datasets to test the mRNA-based two-step SVM classifier. The proteomics-based SVM showed comparable performance to the mRNA-based SVM (Additional file 1:Figure S14), which indicated that features important for predicting mRNA-level miRNA targets are also relevant for predicting protein-level targets. Indeed, when we benchmarked the mRNA-based classifiers against the Selbach and Baek datasets, the SVM outperformed the other seven algorithms in most cases and especially on the ROC_10*n _benchmarks (Table 1; Figure 6). Note that on these two sets, which were true independent test sets for mirTarget2 and therefore should be more representative of the method's performance than the Linsley set, mirTarget2 had similar or slightly lower performance than the two TargetScan versions. These results as well as additional benchmark results (Additional file 1:Figure S15, S16; Table S8, S9) suggest that our two-step SVM approach is also effective at detecting target genes at protein level.

**Table 1:** ROC scores of one transcriptomics and two proteomics datasets

Dataset		SVM	PITA	TScan	miRan	PITAT	TS_C	mirT2	PicTa
Linsley	All	0.81	0.76	0.75	0.55	0.60	0.61	0.63	0.58
	ROC10*n	0.0129	0.0018	0.0173	0.0105	0.0035	0.0170	0.0196	0.0113
	7m+C	0.73	0.61	0.69	0.43	0.57	0.59	0.67	0.57

Selback	All	0.64	0.61	0.61	0.52	0.55	0.55	0.54	0.53
	ROC10*n	0.0253	0.0042	0.0212	0.0079	0.0138	0.0213	0.0231	0.0210
	7m+C	0.71	0.61	0.69	0.42	0.60	0.63	0.60	0.58

Baek	All	0.56	0.56	0.56	0.51	0.52	0.53	0.52	0.52
	ROC10*n	0.0193	0.0046	0.0157	0.0081	0.0148	0.0174	0.0086	0.0131
	7m+C	0.59	0.60	0.62	0.44	0.56	0.61	0.54	0.54

Three benchmarks, All, ROC_10*n_, 7m+C (7mer+Conservation), were performed on one transcripomics (Linsley) and two proteomics (Baek and Selback) datasets. The ROC scores were calculated for eight algorithms, SVM, PITA, TScan (TargetScan), miRan (miRanda), PITAT (PITA Top), TS_C (TargetScan with conserved genes), mirT2 (mirTarget2) and PicTa (PicTar)

### Different criteria of dataset selection do not affect SVM performance

We trained and tested our model with alternative data selection criteria to analyze the influence of data selection on the target prediction power. We verified two data selection criteria, (i) inclusion and exclusion of up regulated targets in the negative record sets, and (ii) different log ratio threshold values to create positive record sets. The results suggest that our SVM approach is very flexible and robust, and it can retain its performance even when trained and tested on datasets created by different parameter settings (see Additional file 1:Supplementary Results).

## Conclusions

We have presented and evaluated a novel two-step SVM-based miRNA target prediction model that recognizes the individual characteristics of functional binding sites and the global characteristics of miRNA-targeted mRNAs. When tested on several different benchmarks, the model generally outperformed other well-known miRNA target prediction algorithms. In particular, the two-step SVM showed a much stronger ability to identify true miRNA targets among genes that all are likely targets with conserved stringent seed sites.

The model relies on several target site and mRNA characteristics and its design is flexible so that it is easy to integrate new characteristics when such are reported. Our current model does not include target site accessibility and conservation information, however, as our tests showed that these were not important features in our model. This result is very important for genome-wide analysis because assessing target site conservation and especially target site accessibility have high computational costs. Consequently, the model can for example be used online for identifying siRNA off-targets.

By modeling miRNA targeting in two steps - recognition of individual target sites and regulation of mRNA - we have incorporated several new characteristics neglected by other tools, such as miRNAs' distance dependence for synergistic regulation. Moreover, through this two-step modeling, we have introduced several features that effectively capture the number and relative strength of individual target sites within target candidates. Our results show that these features are the most important characteristics for identifying miRNA-regulated genes.

A potential limitation is that our model is trained on data from over-expression experiments, which to some extent are affected by interactions with endogenous miRNAs [29]. Including data from Argonaute (AGO) pull-down [32-35] or miRNA knock-down [21-23] experiments could reduce potential bias from such interactions, but may also introduce additional bias. Most of the current AGO pull-down experiments were, for example, done with ectopically expressed and tagged AGO-fusion proteins [32-34]. As for miRNA knock-down, there are currently few datasets available.

Another potential limitation is that our model was trained on data from human cell lines. Although the miRNA regulatory mechanisms appear to be shared in animals, we cannot discount species-specific variation, which may result in the current human-optimized model having sub-optimal performance in, for example, invertebrates. Testing and optimizing the model on high-throughput data from additional species - especially species that are remotely related to humans - would therefore be interesting future work.

Even though our new model represents a significant step towards accurate miRNA target prediction, it is still challenging to achieve very precise target predictions because many miRNAs have spatial, temporal, or cell-type-specific expression. Integrating target predictions with expression profiles of miRNAs and putative targets can be one way to improve predictions; for example, by taking into account miRNA saturation [29], target concentration [36], or expression of RNA binding proteins [37]. Our two-step SVM should be ideal for such analyses, as it has a high sensitivity and better overall performance compared with existing target prediction methods.

## Methods

### Datasets

We downloaded the Jackson [25], Lim [6], Grimson [5], and Linsley [21] datasets from the Gene Expression Omnibus (GEO) database [GEO:GSE5814, GEO:GSE2075, GEO:GSE8501, GEO:GSE6838] [38] and the Birmingham [24] dataset from the ArrayExpress database [ArrayExpress:E-MEXP-668] [39]. We mapped microarray probes to human RefSeq transcripts (hg18) based on GEO and ArrayExpress annotations and downloaded 3' UTR sequences from the University of California, Santa Cruz [40]. Two proteomics datasets, Selbach [23] and Baek [22], were obtained from the original publications along with miRBase and RefSeq IDs. MicroRNA and siRNA sequence data (Additional file 1:Table S6) were obtained from miRBase (release 14.0) [19] and from the original publications, respectively. We used several criteria, including p-values and log intensity ratios (see Additional file 1:Supplementary Methods), to divided the data into positive (down-regulated) and negative genes.

### Construction of target site level features

Potential target sites were identified by nine seed types (8mer, 7mer-A1, 7mer-m8, 6mer, GUM, GUT, LP, BM and BT) in human 3' UTRs between position 15 from the stop codon and the 3' end. Partially overlapping sites were only allowed among the sites with 8mer, 7mer-A1, 7mer-m8, and 6mer. The precedence of selecting a target site among overlapping sites was defined as 8mer = 7mer-A1 = 7mer-m8 = 6mer > GUM > GUT > LP > BM > BT. Consequently, if a GUM and GUT site overlapped, the GUM site was selected. Target sites and their corresponding miRNAs were aligned using the emboss needle software [41]; see Additional file 1:Table S7 for needle parameters.

We used 24 target site level features (Additional file 1:Table S1). Site accessibility features were created by the PITA software (version 3) by considering either no flanking regions or flanking regions consisting of 3 nts upstream and 15 nts downstream of the site [13]. Evolutionary conservation scores were calculated from phastCons 44, phyloP 44, and multiz 17-way [42-44] scores downloaded from UCSC. The phastCons 44 scores were used to calculate the average score of the whole target site (position 1 to 20), whereas the phyloP 44 scores were used as the conservation scores of the seed site (position 1 to 8). All feature values were normalized into values ranged between -1 and 1 by a linear transform based on the feature's minimum and maximum values among the sites in the training set.

### Construction of mRNA level features

Construction of mRNA level training data was done in three steps. First, discriminant values, which are the output values from SVM classification, were obtained from the target site level 10-fold cross-validation test sets. In our model, these discriminant values represented the predicted regulatory strength of each target site. Second, discriminant values were matched to mRNAs such that an mRNA with for example three miRNA target sites would have three discriminant values - one value for each site. Third, the discriminant values and other information about the target sites and the 3' UTR were used to calculate 17 mRNA level features (Additional file 1:Table S3).

SVMs require fixed-length feature vectors, but the number of target sites varies between mRNAs. Consequently, to represent the number and predicted strength of individual target sites within a given mRNA, we used a feature representation that encoded the overall distribution of target site level discriminant values. For each mRNA, this feature consisted of 16 values that counted the number of target site discriminant values that fell within given percentile ranges of the overall discriminant value distribution. Specifically, two feature values counted the number of upper and lower extreme discriminant values greater than and less than two standard deviations away from the distribution mean. The remaining 14 feature values counted the number of discriminant values falling within the bins defined by the 25.00, 43.75, 57.81, 68.36, 76.27, 82.20, 86.65, 89.99, 92.49, 94.37, 95.78, 97.19, 98.6, and 100 percentiles when the upper and lower extreme values were removed from the discriminant value distribution. We used these percentile thresholds because we expected a high resolution at the upper tail of the discriminant value distribution to be useful for predictions. Supporting this hypothesis, replacing these 14 thresholds with 14 uniformly spaced thresholds gave markedly poorer SVM performance (data not shown).

### Target site/mRNA level training

We used the PyML library (Version 0.72) [45] for SVM training, 10-fold cross-validation, classification, and evaluation, and the R package ROCR [46] to plot Receiver Operating Characteristics (ROC) curves and to calculate the area under the ROC curve (AUC).

To optimize our model, we tested a linear kernel and three non-linear kernels - Gaussian, homogeneous polynomial, and inhomogeneous polynomial. In addition to the four kernel types, three parameters - cost factor (C), gamma (*γ*), and degree (*d*) - were tested. The parameter ranges were C = 2^(2*n *- 5) ^(0 ≤ *n *≤ 11), *γ *= 2^(2*n *- 13) ^(0 ≤ *n *≤ 10), and *d *= *n *(2 ≤ *n *≤ 8) as previously recommended [47].

### Data retrieval for benchmarks

Prediction data were downloaded from the PITA, TargetScan, MicroCosm, miRDB, and PicTar websites (see Additional file 1:Supplementary Methods).

## Authors' contributions

Both authors contributed to the underlying ideas of the method and the analysis. TS implemented the model. The initial manuscript draft was written by TS, and refined by PS. Both authors read and approved the final manuscript.

## Supplementary Material

Additional file 1 **Supplementary information**. The file contains five sections of supplementary information, and the sections are: Supplementary Methods (3 subsections), Supplementary Results (6 subsections), Supplementary Tables (15 tables), Supplementary Figures (22 figures), and the Reference. Click here for file

## References

[B1] Bartel DP MicroRNAs: genomics, biogenesis, mechanism, and function Cell 2004 116 2 281 297 10.1016/S0092-8674(04)00045-5 14744438

[B2] Ambros V The functions of animal microRNAs Nature 2004 431 7006 350 355 10.1038/nature02871 15372042

[B3] Stefani G Slack FJ Small non-coding RNAs in animal development Nat Rev Mol Cell Biol 2008 9 3 219 230 10.1038/nrm2347 18270516

[B4] Rajewsky N microRNA target predictions in animals Nat Genet 2006 38 Suppl S8 13 10.1038/ng1798 16736023

[B5] Grimson A Farh KK Johnston WK Garrett-Engele P Lim LP Bartel DP MicroRNA targeting specificity in mammals: determinants beyond seed pairing Mol Cell 2007 27 1 91 105 10.1016/j.molcel.2007.06.017 17612493 PMC3800283

[B6] Lim LP Lau NC Garrett-Engele P Grimson A Schelter JM Castle J Bartel DP Linsley PS Johnson JM Microarray analysis shows that some microRNAs downregulate large numbers of target mRNAs Nature 2005 433 7027 769 773 10.1038/nature03315 15685193

[B7] Stark A Brennecke J Bushati N Russell RB Cohen SM Animal MicroRNAs confer robustness to gene expression and have a significant impact on 3'UTR evolution Cell 2005 123 6 1133 1146 10.1016/j.cell.2005.11.023 16337999

[B8] Bartel DP MicroRNAs: target recognition and regulatory functions Cell 2009 136 2 215 233 10.1016/j.cell.2009.01.002 19167326 PMC3794896

[B9] Saito T Sætrom P MicroRNAs-targeting and target prediction New Biotechnology 2010 27 3 243 249 10.1016/j.nbt.2010.02.016 20219708

[B10] Lewis BP Shih I-h Jones-Rhoades MW Bartel DP Burge CB Prediction of mammalian microRNA targets Cell 2003 115 7 787 798 10.1016/S0092-8674(03)01018-3 14697198

[B11] Majoros WH Ohler U Spatial preferences of microRNA targets in 3' untranslated regions BMC Genomics 2007 8 152 10.1186/1471-2164-8-152 17555584 PMC1904200

[B12] Gaidatzis D van Nimwegen E Hausser J Zavolan M Inference of miRNA targets using evolutionary conservation and pathway analysis BMC Bioinformatics 2007 8 69 10.1186/1471-2105-8-69 17331257 PMC1838429

[B13] Kertesz M Iovino N Unnerstall U Gaul U Segal E The role of site accessibility in microRNA target recognition Nat Genet 2007 39 10 1278 1284 10.1038/ng2135 17893677

[B14] Long D Lee R Williams P Chan CY Ambros V Ding Y Potent effect of target structure on microRNA function Nat Struct Mol Biol 2007 14 4 287 294 10.1038/nsmb1226 17401373

[B15] Saetrom P Heale BSE Snøve O Aagaard L Alluin J Rossi JJ Distance constraints between microRNA target sites dictate efficacy and cooperativity Nucleic Acids Res 2007 35 7 2333 2342 10.1093/nar/gkm133 17389647 PMC1874663

[B16] Enright AJ John B Gaul U Tuschl T Sander C Marks DS MicroRNA targets in Drosophila Genome Biol 2003 5 1 R1 10.1186/gb-2003-5-1-r1 14709173 PMC395733

[B17] Krek A Grun D Poy MN Wolf R Rosenberg L Epstein EJ MacMenamin P da Piedade I Gunsalus KC Stoffel M Combinatorial microRNA target predictions Nat Genet 2005 37 5 495 500 10.1038/ng1536 15806104

[B18] Vapnik VN Statistical Learning Theory 1998 Wiley, New York

[B19] Griffiths-Jones S Saini HK van Dongen S Enright AJ miRBase: tools for microRNA genomics Nucleic Acids Res 2008 36 Database D154 158 17991681 10.1093/nar/gkm952 PMC2238936

[B20] Wang X El Naqa IM Prediction of both conserved and nonconserved microRNA targets in animals Bioinformatics 2008 24 3 325 332 10.1093/bioinformatics/btm595 18048393

[B21] Linsley PS Schelter J Burchard J Kibukawa M Martin MM Bartz SR Johnson JM Cummins JM Raymond CK Dai H Transcripts targeted by the microRNA-16 family cooperatively regulate cell cycle progression Mol Cell Biol 2007 27 6 2240 2252 10.1128/MCB.02005-06 17242205 PMC1820501

[B22] Baek D Villén J Shin C Camargo FD Gygi SP Bartel DP The impact of microRNAs on protein output Nature 2008 455 7209 64 71 10.1038/nature07242 18668037 PMC2745094

[B23] Selbach M Schwanhausser B Thierfelder N Fang Z Khanin R Rajewsky N Widespread changes in protein synthesis induced by microRNAs Nature 2008 455 7209 58 63 10.1038/nature07228 18668040

[B24] Birmingham A Anderson EM Reynolds A Ilsley-Tyree D Leake D Fedorov Y Baskerville S Maksimova E Robinson K Karpilow J 3'UTR seed matches, but not overall identity, are associated with RNAi off-targets Nat Methods 2006 3 3 199 204 10.1038/nmeth854 16489337

[B25] Jackson AL Burchard J Schelter J Chau BN Cleary M Lim L Linsley PS Widespread siRNA "off-target" transcript silencing mediated by seed region sequence complementarity RNA 2006 12 7 1179 1187 10.1261/rna.25706 16682560 PMC1484447

[B26] Hamilton AJ Baulcombe DC A species of small antisense RNA in posttranscriptional gene silencing in plants Science 1999 286 5441 950 952 10.1126/science.286.5441.950 10542148

[B27] Zamore PD Tuschl T Sharp PA Bartel DP RNAi: double-stranded RNA directs the ATP-dependent cleavage of mRNA at 21 to 23 nucleotide intervals Cell 2000 101 1 25 33 10.1016/S0092-8674(00)80620-0 10778853

[B28] Doench JG Petersen CP Sharp PA siRNAs can function as miRNAs Genes Dev 2003 17 4 438 442 10.1101/gad.1064703 12600936 PMC195999

[B29] Khan AA Betel D Miller ML Sander C Leslie CS Marks DS Transfection of small RNAs globally perturbs gene regulation by endogenous microRNAs Nat Biotechnol 2009 27 6 549 555 19465925 10.1038/nbt.1543 PMC2782465

[B30] Betel D Koppal A Agius P Sander C Leslie C Comprehensive modeling of microRNA targets predicts functional non-conserved and non-canonical sites Genome Biol 2010 11 8 R90 10.1186/gb-2010-11-8-r90 20799968 PMC2945792

[B31] Gribskov M Robinson NL Use of receiver operating characteristic (ROC) analysis to evaluate sequence matching Comput Chem 1996 20 1 25 33 10.1016/S0097-8485(96)80004-0 16718863

[B32] Karginov FV Conaco C Xuan Z Schmidt BH Parker JS Mandel G Hannon GJ A biochemical approach to identifying microRNA targets Proc Natl Acad Sci USA 2007 104 49 19291 19296 10.1073/pnas.0709971104 18042700 PMC2148283

[B33] Hafner M Landthaler M Burger L Khorshid M Hausser J Berninger P Rothballer A Ascano M Jr Jungkamp AC Munschauer M Transcriptome-wide identification of RNA-binding protein and microRNA target sites by PAR-CLIP Cell 2010 141 1 129 141 10.1016/j.cell.2010.03.009 20371350 PMC2861495

[B34] Hendrickson DG Hogan DJ Herschlag D Ferrell JE Brown PO Systematic identification of mRNAs recruited to argonaute 2 by specific microRNAs and corresponding changes in transcript abundance PLoS One 2008 3 5 e2126 10.1371/journal.pone.0002126 18461144 PMC2330160

[B35] Chi SW Zang JB Mele A Darnell RB Argonaute HITS-CLIP decodes microRNA-mRNA interaction maps Nature 2009 460 7254 479 486 19536157 10.1038/nature08170 PMC2733940

[B36] Arvey A Larsson E Sander C Leslie CS Marks DS Target mRNA abundance dilutes microRNA and siRNA activity Mol Syst Biol 2010 6 363 10.1038/msb.2010.24 20404830 PMC2872614

[B37] Jacobsen A Wen J Marks DS Krogh A Signatures of RNA binding proteins globally coupled to effective microRNA target sites Genome Res 2010 20 8 1010 1019 10.1101/gr.103259.109 20508147 PMC2909566

[B38] Barrett T Troup DB Wilhite SE Ledoux P Rudnev D Evangelista C Kim IF Soboleva A Tomashevsky M Edgar R NCBI GEO: mining tens of millions of expression profiles-database and tools update Nucleic Acids Res 2007 35 Database D760 D765 10.1093/nar/gkl887 17099226 PMC1669752

[B39] Parkinson H Kapushesky M Kolesnikov N Rustici G Shojatalab M Abeygunawardena N Berube H Dylag M Emam I Farne A ArrayExpress update-from an archive of functional genomics experiments to the atlas of gene expression Nucleic Acids Res 2009 37 Database D868 872 10.1093/nar/gkn889 19015125 PMC2686529

[B40] UCSC Genome Browser http://genome.ucsc.edu

[B41] Rice P Longden I Bleasby A EMBOSS: the European Molecular Biology Open Software Suite Trends Genet 2000 16 6 276 277 10.1016/S0168-9525(00)02024-2 10827456

[B42] Blanchette M Kent WJ Riemer C Elnitski L Smit AF Roskin KM Baertsch R Rosenbloom K Clawson H Green ED Aligning multiple genomic sequences with the threaded blockset aligner Genome Res 2004 14 4 708 715 10.1101/gr.1933104 15060014 PMC383317

[B43] Pollard KS Hubisz MJ Rosenbloom KR Siepel A Detection of nonneutral substitution rates on mammalian phylogenies Genome Res 2010 20 1 110 121 10.1101/gr.097857.109 19858363 PMC2798823

[B44] Siepel A Bejerano G Pedersen JS Hinrichs AS Hou M Rosenbloom K Clawson H Spieth J Hillier LW Richards S Evolutionarily conserved elements in vertebrate, insect, worm, and yeast genomes Genome Res 2005 15 8 1034 1050 10.1101/gr.3715005 16024819 PMC1182216

[B45] PyML library http://pyml.sourceforge.net/

[B46] Sing T Sander O Beerenwinkel N Lengauer T ROCR: visualizing classifier performance in R Bioinformatics 2005 21 20 3940 3941 10.1093/bioinformatics/bti623 16096348

[B47] A practical guide to support vector classification http://www.csie.ntu.edu.tw/~cjlin/papers/guide/guide.pdf

