# Supplementary material for: A two-step site and mRNA-level model for predicting microRNA targets
Source: BMC Bioinformatics. 2010 Dec 31;11:612. doi: 10.1186/1471-2105-11-612 (PMC3019229; doi:10.1186/1471-2105-11-612)
Supplement: Additional file 1 — Supplementary information. The file contains five sections of supplementary information, and the sections are: Supplementary Methods (3 subsections), Supplementary Results (6 subsections), Supplementary Tables (15 tables), Supplementary Figures (22 figures), and the Reference. [file 1471-2105-11-612-S1.PDF]

Supplementary information

A two-step site and mRNA-level model for predicting microRNA targets

Takaya Saito and Pål Sætrom

Contents

Supplementary Methods

Data partition into positive (down-regulated) and negative genes
Data retrieval for benchmarks
Performance evaluation on independent dataset

Supplementary Results

Analysis of target site level training data: Selection of seed types influences the coverage of potential target sites
Prediction of targets with optimal distance sites: Optimal distance is effectively incorporated in our SVM prediction
Site accessibility and sequence conservation: Explicit usage of site accessibility and sequence conservation do not improve SVM prediction
Classification vs. Regression: Support vector classification (SVC) performs better than support vector regression (SVR)
SVM performance on proteomics data: Additional benchmarks show the SVM classification ability at protein level
Dataset selection: Different parameters of selecting training or test data do not affect prediction ability

Supplementary Tables

Table S1. Target site level features
Table S2. Analysis of target site level feature influence
Table S3. mRNA level features
Table S4. Analysis of mRNA level feature influence
Table S5. Benchmarks on the Linsley dataset
Table S6. mRNA/siRNA sequences from five microarray experiments
Table S7. Parameters used for the Needle software
Table S8. Benchmarks on the Selbach dataset
Table S9. Benchmarks on the Baek dataset
Table S10. Benchmarks on the Linsley dataset – log ratio: -0.1
Table S11. Benchmarks on the Linsley dataset – log ratio: -0.3
Table S12. Benchmarks on the Selbach dataset – log ratio: -0.1
Table S13. Benchmarks on the Selbach dataset – log ratio: -0.3
Table S14. Benchmarks on the Baek dataset – log ratio: -0.1
Table S15. Benchmarks on the Baek dataset – log ratio: -0.3

Supplementary Figures

Figure S1. 10-fold cross-validation of target site level classifiers trained on three microarray datasets
Figure S2. Density plot for the number of potential target sites divided by 3'UTR length
Figure S3. Comparison between two SVM trainings with or without explicit addition of negative records
Figure S4. 10-fold cross-validation of mRNA level classifiers trained on three microarray datasets
Figure S5. Three method-specific benchmarks of 8 different algorithms on the Linsley dataset
Figure S6. siRNA benchmarks on the Jackson dataset

Figure S7. miRNA benchmarks on the Lim dataset
Figure S8. siRNA benchmarks on the Birmingham dataset
Figure S9. miRNA benchmarks on Grimson dataset
Figure S10. Targets with optimal distance sites show higher average discriminant value than other three target types
Figure S11. Adding site accessibility and sequence conservation features did not affect target prediction accuracy
Figure S12. SVC performs better than SVR at both target site and mRNA levels
Figure S13. SVM performance is similar between the training set with up-regulated genes and the one without up-regulated genes
Figure S14. Two-step SVM retains the performance when trained with proteomics data
Figure S15. Benchmarks on the Selbach dataset
Figure S16. Benchmarks on the Baek dataset
Figure S17. Benchmarks on the Linsley dataset with positive record threshold by log ratio -0.1
Figure S18. Benchmarks on the Linsley dataset with positive record threshold by log ratio -0.3
Figure S19. Benchmarks on the Selbach dataset with positive record threshold by log ratio -0.1
Figure S20. Benchmarks on the Selbach dataset with positive record threshold by log ratio -0.3
Figure S21. Benchmarks on the Baek dataset with positive record threshold by log ratio -0.1
Figure S22. Benchmarks on the Baek dataset with positive record threshold by log ratio -0.3

References

Supplementary Methods

Data partition into positive (down-regulated) and negative genes

Down-regulated (positive) genes of GEO datasets were defined as records with p-value < 0.001 and log intensity ratio < -0.3 to obtain strongly down-regulated genes. Down-regulated genes for the Birmingham dataset were obtained directly from the published data (Birmingham, Anderson et al. 2006).

Some microarray records contained different results for the same combination of miRNA/siRNA and mRNA; for example, there were five records of a miRNA on the same mRNA, and four of them were regarded as down-regulated but one of them were unaffected. In this case, all records that belonged to this combination were disregarded to enhance the quality.

Negative records of GEO datasets consisted of both unaffected and up-regulated genes. Unaffected genes were defined as records with p-value > 0.3 and log intensity ratio between -0.3 and 0.3, whereas up-regulated genes were defined as records with p-value < 0.001 and log intensity ratio > 0.3. Negative records for the Birmingham dataset were defined as all RefSeq transcripts except the positive records.

As a final filter, all miRNA:mRNA pairs that had either very high (>1.25) or very low (<-1.5) log intensity values in the control experiments were eliminated to reduce potential noise form extreme expression values.

All the positive records were used in the training dataset, whereas negative records were randomly selected to meet a total record number of 20000, unless otherwise specified. A maximum total record number of 20000 was used due to computational time and memory space constraints.

For the test data, positive records were selected as for the training data, whereas all the remaining records were used as negative records.

For the Linsley dataset (Linsley, Schelter et al. 2007), down-regulated genes were defined as records with p-value < 0.001 and log intensity ratio < -0.2.

For the Selbach and Baek datasets, down-regulated genes (positive) were defined as records with log ratio values < -0.2, whereas unaffected genes (negative) were defined as log ratio values > 0. The records that do not fit in these criteria were not used in this study.

Data retrieval for benchmarks

Prediction data were downloaded from the Segal Lab site (http://genie.weizmann.ac.il/pubs/mir07) for PITA All and PITA Top, the TargetScanHuman 5.1 site (http://www.targetscan.org) for TargetScan and TargetScan with conserved genes, the MicroCosm Targets Version 5 site (http://www.ebi.ac.uk/enright-srv/microcosm) for miRanda, the miRDB site (http://mirdb.org) for mirTarget2, and the PicTar WEB INTERFACE site (http://pictar.mdc-berlin.de) for PicTar. Several methods provide only target site level scores for download, and, in that case, summation methods showed in the web sites or in the original publications were applied to obtain scores for the mRNA level. For siRNA predictions, PITA and TargetScan executable programs were downloaded and used to calculate prediction scores.

Performance evaluation on independent dataset

Six dataset – “ROC with All genes”, “ROC_{10*n}”, “ROC with 7mer + Conservation”, and three method-specific datasets for TargetScan, miRanda, and PicTar – were created

for evaluation. The “ROC with All genes” dataset was comprised of all the records from the microarray dataset.

“ROC_{10*n}” was based on the standard ROC_{50} (Gribskov and Robinson 1996) benchmark. The ROC_{50} benchmark score is the area under the ROC curve until 50 negatives are found. Consequently, the “ROC_{10*n}” benchmark datasets consisted of all the true positives found in the microarray experiments and the 10*n first negatives found by each method benchmarked.

The “ROC with 7mer + Conservation” dataset was comprised of genes with conserved 8mer or 7mer-m8 or 7mer-A1 sites. Sites were defined as conserved if the average phyloP 44 scores in the seed region were > 0.9.

The three method-specific datasets for TargetScan, miRanda, and PicTar were generated by only using the records from the microarray experiments that had corresponding predictions for each method.

Supplementary Results

Analysis of target site level training data: Selection of seed types influences the coverage of potential target sites

Current methods for miRNA target prediction base their analyses on identifying so-called seed sites in 3' UTR. The definition of seed types varies from one prediction algorithm to another (Gaidatzis, van Nimwegen et al. 2007; Grimson, Farh et al. 2007; Hammell, Long et al. 2008), however, and this heavily influences the coverage of potential target sites. Lower coverage means that predictions may lack many true positive sites, whereas higher coverage may result in poor accuracy because of too many false positive sites.

Based on four different microarray datasets - Birmingham (Birmingham, Anderson et al. 2006), Jackson (Jackson, Burchard et al. 2006), Lim (Lim, Lau et al. 2005), and Grimson (Grimson, Farh et al. 2007), we identified a set of 823 genes that were down-regulated in response to artificial miRNA or siRNA transfection, or up-regulated in response to miRNA inhibition (see Supplementary Methods). This positive set of genes constituted 0.14% of the 571570 potential miRNA:gene interactions assayed in the microarray experiments. Searching for seed sites among the positive genes identified that 347 and 510 genes contained stringent and all types of seed sites. Thus, basing miRNA target predictions on stringent seeds or all seed types would cover 42% or 62% of the positive genes. The corresponding numbers for the negative set of 570747 genes were 128254 and 31277. Consequently, the predictive accuracy, as measured by the positive predictive value (PPV = True positive predictions / All positive predictions), was almost three times greater for stringent seeds (PPV = 1.1%) than for all seed types (PPV = 0.40%). Even so, this showed that predicting miRNA targets purely based on identifying stringent seed sites would give too many false positive predictions to be viable in practice.

Several approaches have shown that other target-related features besides the seed type and additional data such as miRNA and mRNA expression profiles can improve predictive accuracy (Huang, Babak et al. 2007; Sales, Coppe et al. 2010). We therefore chose to include all seed types to achieve high coverage in the training data and then improve the accuracy through two-step SVM training.

Prediction of targets with optimal distance sites: Optimal distance is effectively incorporated in our SVM prediction

We were interested to see how well the features of optimal distances were incorporated into our algorithm. Our model uses two mRNA features that represent optimal distances: (i) the number of sites that have other neighboring sites within 13-46 nt and (ii) the number of sites that have other neighboring sites within 17-25 nt. Furthermore, one target site level feature, "distance to the next target" could be effective to recognize the targets with optimal distance. However, these features showed little contribution when the influence of both target site and mRNA level features was analyzed (Tables S2 and S4). Therefore, we applied a different approach as to see the difference of prediction power between the target genes with optimal distance and other target genes.

First, we categorized all potential targets into four different target types, which were defined by the number of potential target sites, seed types, and existence of optimal distance sites within the gene's 3' UTR. "Single non-stringent" and "Single stringent" were targets that had only one "non-stringent" and "stringent" seed site, whereas "Multiple without optimal distance" and "Multiple with optimal distance" were targets that had multiple sites where none or at least one pair of sites were within . Then, we checked the mRNA level classifier's distributions of discriminant values for these four target types. The resulting box plots (Fig. S9) show that for each target type, the mRNA-level SVM clearly separates between the positive (down-regulated) and negative (non-down-regulated) targets. Moreover, targets with multiple sites had higher discriminant values than targets with single stringent and single non-stringent sites, and the targets with optimally spaced sites had the highest discriminant values. Thus, site distance contributed positively to the SVM's predictions.

Site accessibility and sequence conservation: Explicit usage of site accessibility and sequence conservation do not improve SVM prediction

Although target site accessibility is important for both miRNA and siRNA targeting (Kertesz, Iovino et al. 2007; Long, Lee et al. 2007), it is unclear how useful computational predictions of target site accessibility are for genome-wide miRNA

target analyses (Baek, Villén et al. 2008; Hammell, Long et al. 2008). Similarly, even though many target prediction tools rely on conserved sequences, as these indicate conserved function and targeting, sequences with little or no evidence of conservation can still be functional target sites (Sethupathy, Megraw et al. 2006). Moreover, calculating target site accessibility and conservation features demand much higher computational power and resources than calculating the other features. Therefore, we constructed classifiers without using the site accessibility and sequence conservation features to evaluate their importance in our SVM model.

To evaluate the classifier without of site accessibility and sequence conservation features, we compared the ROC scores of three mRNA level classifiers that used the same mRNA level features, but three different target site classifiers; that is, classifiers (i) without site accessibility and conservational information, (ii) with site accessibility, and (iii) with conservational information. The classifiers were then tested on the independent dataset.

All the results from the 10-fold cross-validation and independent tests showed very little difference among the three classifiers (Fig. S10). Although theoretically sound, the computationally intensive accessibility predictions could therefore, in practice, be eliminated from the SVM classifier. In addition, although many miRNA target sites appear to be well conserved, the small influence of the conservation-related features on SVM performance indicate that many non-conserved miRNA targets exist. This is consistent with the results of the benchmarks of the 8 existing miRNA target prediction methods. Consequently, the proportion of non-conserved miRNA target sites in the set of all potential target sites seems too large to be ignored. Therefore, we opted to train our SVM classifiers without using site accessibility and sequence conservation.

Classification vs. Regression: Support vector classification (SVC) performs better than support vector regression (SVR)

The microarray data used in our experiments measured actual changes in gene expression in response to miRNA or siRNA over-expression. Consequently, even though we treated miRNA target prediction as a classification problem, target prediction could also be solved as a regression problem. To test whether treating target prediction as a regression problem would further improve our predictions, we

used the PyML library to train SVR models to fit the log ratio values from the Jackson, Lim, and Grimson microarray experiments. We did not use the Birmingham dataset because pre-processed log ratio values were not available. The SVR models used the same two step approach and the same features as our previous SVC models. The same optimization approach as we used for the SVC models showed that a linear kernel with default parameters gave the best predictions at both the target site and mRNA level. This model selection often resulted in over-fitting or no convergence, however, hence requiring more analysis time than SVC to find an optimal kernel. Moreover, the SVR target site and mRNA level models showed lower prediction performance at the 10-fold cross-validation and independent data set than the SVC models did (Fig. S11). Thus, when the input is based on features derived from the target sequence, classification models are as good or better at predicting miRNA or siRNA target gene expression fold changes as are regression models.

Dataset selection: Different parameters for constructing training or test data do not affect SVM prediction performance

It is important to verify that our SVM approach is still effective when we select different positive (down-regulated) and negative (non-down-regulated) datasets for both training and test because it proves that our SVM approach is not optimized only for a certain parameter criteria of data selection. We generated seven different datasets; one for training and six for independent test sets to further investigate SVM prediction power with different data selection parameters.

Two major steps applied to create datasets in our study were; (i) all microarray datasets were separated into three groups; down-regulated, unaffected and up-regulated, depending of the log ratio values, and (ii) positive data were selected from down-regulated genes, whereas negative records were from both unaffected and up-regulated genes. However, some miRNAs are known to potentially up-regulate genes. The mechanism of this up-regulation is unknown, and the features of up-regulated target sites/genes are possibly quite different from those of miRNAs involved in down-regulation. To check the influence of involving these up-regulated genes in our SVM training sets, we constructed a dataset without up-regulated genes, and trained and tested it on the independent datasets. The 10-fold cross-validation ROC plots for both target site and mRNA level classifiers trained without

up-regulated genes (Fig. S12 A, C) were almost identical to those trained with up-regulated genes (Fig. S12 E, G). The prediction power was also retained when the classifiers were tested on the Linsley dataset (Fig. S12 B, D) and compared with those trained with up-regulated genes (Fig. S12 F, H).

Moreover, we generated six independent subsets for one transcriptomics (Linsley) and two proteomics (Selbach and Baek) experiments. Two different positive record datasets defined by log-ratio values <-0.1 and <-0.3 were created for each experiment, resulting in six independent datasets altogether. We performed benchmarks on these datasets and compared with the dataset defined by the log-ratio values <-0.2, which had been used as the default value for all the other benchmarks. The number of positive records detected by all of the eight algorithms in the benchmarks was higher in the log ratio <-0.1 sets; Linsley (Table S10), Selback (Table S12), and Baek (Table S14), and lower in the log ratio <-0.3 sets; Linsley (Table S11), Selback (Table S13), and Baek (Table S15), as expected. The ROC scores from the benchmarks show that SVM outperforms other algorithms in most cases regardless of different parameter criteria of data selection. We also analyzed the ROC curves of the benchmarks to check the trends among the log ratio <-0.2 sets; Linsley (Fig. 5, S4), Selback (Fig. 6, Fig. 14) and Baek (Fig.6, Fig. 15), the log ratio <-0.1 sets; Linsley (Fig. S16), Selback (Fig. 18) and Baek (Fig. 20), and the log ratio <-0.3 sets; Linsley (Fig. S17), Selback (Fig. 19) and Baek (Fig. 21). All ROC curves showed very similar trends among different positive record sets. More importantly, our SVM approach retained its prediction power even when it was tested on the log ratio <-0.3 and <-0.1 sets.

These results indicate that our SVM prediction power is not affected when trained with or without up-regulated genes, or tested on strongly down-regulated pairs (log ratio <-0.3) as well as moderately down-regulated pairs (log ratio <-0.1).

Supplementary Tables

Table S1. Target site level features.

	Feature	Vector size[a]	Site[b]	Consv[c]	Incl[d]
1	Seed Type	9			○
2	Similarity (1-20 nt)	1			○
3	Similarity in the 5' portion (1-9 nt)	1			○
4	Similarity in the 3' portion (>10 nt)	1			○
5	Similarity in the 3' portion (position 13,14,15,16)	1			○
6	A:U rich context in the 30nt upstream	30			○
7	A:U rich context in the 30nt downstream	30			○
8	Relative site position (position / 3' UTR length)	1			○
9	Distance to the next-nearest-neighbor site	1			○
10	Site accessibility: dGduplex	1	○		
11	Site accessibility: dGopen	1	○		
12	Site accessibility: ddG	1	○		
13	Site accessibility with 3/15 flank: dGopen	1	○		
14	Site accessibility with 3/15 flank: ddG	1	○		
15	20nt site matches (Match:1, AU:0.4, None-match:0)	20			○
16	Pos 1 nucleotide of the target site (A:1, UCG:0)	1			○
17	multiz17way score for the seed site	1		○	
18	multiz17way score for the target site	1		○	
19	PhastCons44way scores for the target site	1		○	
20	PhastCons44wayMammal scores for the target site	1		○	
21	PhastCons44wayPrimate scores for the target site	1		○	
22	PhyloP44ways scores for the seed site	8		○	
23	PhyloP44waysMammal scores for the seed site	8		○	
24	PhyloP44waysPrimate scores for the seed site	8		○	

[a]Vector size used in SVM training. [b]Site accessibility features. ○: Yes, blank: No. [c]Sequence conservation features. ○: Yes, Blank: No. [d]Features included in the final version of our classifier. ○: included features, blank: features excluded during optimization steps.

Table S2. Analysis of target site level feature influence.

Rank	ROC	ROC diff[a]	Removed feature
1	0.6734	0.0317	Seed Type
2	0.6958	0.0093	20nt site matches (Match:1, AU:0.4, None-match:0)
3	0.6998	0.0053	A:U rich context in the 30nt upstream
4	0.7047	0.0004	PhastCons44wayMammal scores for the target site
5	0.7047	0.0003	Similarity in the 3' portion (>10 nt)
6	0.7048	0.0002	Relative site position (position / 3'UTR length)
7	0.7049	0.0001	Similarity in the 5' portion (1-9 nt)
8	0.7050	0.0000	Site accessibility: dGopen
9	0.7050	0.0000	Distance to the next-nearest-neighbor site
10	0.7050	0.0000	Site accessibility: dGduplex
11	0.7050	0.0000	Site accessibility: ddG
12	0.7050	0.0000	Site accessibility with 3/15 flank: dGopen
13	0.7050	0.0000	Site accessibility with 3/15 flank: ddG
14	0.7051	-0.0001	Similarity in the 3' portion (position 13,14,15,16)
15	0.7053	-0.0002	PhyloP44ways scores for the seed site
16	0.7054	-0.0003	Similarity (1-20 nt)
17	0.7054	-0.0004	multiz17way score for the seed site
18	0.7056	-0.0006	Pos 1 nucleotide of the target site (A:1, UCG:0)
19	0.7057	-0.0006	A:U rich context in the 30nt downstream
20	0.7059	-0.0008	multiz17way score for the target site
21	0.7060	-0.0009	PhastCons44wayPrimate scores for the target site
22	0.7064	-0.0014	PhyloP44waysMammal scores for the seed site
23	0.7068	-0.0017	PhastCons44way scores for the target site
24	0.7081	-0.0030	PhyloP44waysPrimate scores for the seed site

[a]ROC diff shows the difference of ROC scores between the classifier trained with all the features (0.7050) and classifiers trained with one feature removed.

Table S3. mRNA level features.

	Feature	Vector size[a]	Incl[b]
1	3'UTR Length	1	○
2	Number of target sites	1	○
3	Total discriminant value / 3' UTR length	1	○
4	Number of 8mer sites	1	○
5	Number of 7mer-m8 sites	1	○
6	Number of 7mer-A1 sites	1	○
7	Number of 6mer sites	1	○
8	Number of GUM sites	1	○
9	Number of GUT sites	1	○
10	Number of LP sites	1	○
11	Number of BT sites	1	○
12	Number of BM sites	1	○
13	Distribution bins of discriminant values	16	○
14	Site counts within optimal distance 14-46 nt	1	○
15	Site counts within optimal distance 17-25 nt	1	○
16	Site number flags (1 or 2-7 or >8)	3	○
17	Total discriminant value	1	○

[a]Vector size used in SVM training. [b]Features included in the final version of our classifier. ○: included features, blank: features excluded during optimization steps.

Table S4. Analysis of mRNA level feature influence.

Rank	ROC	ROC diff[a]	Removed feature
1	0.7565	0.0432	Distribution bins of discriminant values
2	0.7904	0.0093	Number of GUM sites
3	0.7971	0.0026	Site number flags (1 or 2-7 or >8)
4	0.7978	0.0019	Total discriminant value / 3' UTR length
5	0.7981	0.0016	Number of 7mer-m8 sites
6	0.7984	0.0013	Site counts within optimal distance 17-25 nt
7	0.7986	0.0011	Number of BT sites
8	0.7994	0.0003	Number of 6mer sites
9	0.7995	0.0002	Number of 7mer-A1 sites
10	0.7996	0.0001	Total discriminant value
11	0.7996	0.0001	3' UTR Length
12	0.7996	0.0001	Number of GUT sites
13	0.7997	0.0000	Number of BM sites
14	0.7997	0.0000	Number of 8mer sites
15	0.7999	-0.0002	Number of LP sites
16	0.8003	-0.0006	Site counts within optimal distance 14-46 nt
17	0.8009	-0.0012	Number of target sites

[a]ROC diff shows the difference of ROC scores between the classifier trained with all the features (0.7997) and classifiers trained with one feature removed.

Table S5. Benchmarks on the Linsley dataset.

Benchmark	Method	# of targets[a]		ROC[b]
		Positive	Negative	
All genes	All	667	158374	
	SVM	564	69037	0.81
	PITA	539	55953	0.76
	TargetScan	400	21228	0.75
	miRanda	93	6641	0.55
	PITA_top	147	4867	0.6
	TargetScan_consv	174	5634	0.61
	mirTarget2	191	3386	0.63
	PicTar	124	3826	0.58
ROC_{10*n}	All	667	90	
	SVM	16	90	0.0168
	PITA	1	90	0.0058
	TargetScan	13	90	0.0267
	miRanda	11	90	0.0144
	PITA_top	2	90	0.0076
	TargetScan_consv	14	90	0.0209
	mirTarget2	19	90	0.0235
	PicTar	17	90	0.0151
7mer + Conservation	All	353	15823	
	SVM	339	12731	0.73
	PITA	339	13230	0.61
	TargetScan	310	10715	0.69
	miRanda	93	6641	0.43
	PITA_top	147	4867	0.57
	TargetScan_consv	174	5634	0.59
	mirTarget2	191	3386	0.67
	PicTar	124	3826	0.57
TargetScan	All	174	5658	
	SVM	168	5022	0.67
	PITA	174	5440	0.56
	TargetScan	174	5634	0.65
	miRanda	36	827	0.53
	PITA_top	119	3527	0.56
	TargetScan_consv	174	5634	0.65
	mirTarget2	109	1926	0.66
	PicTar	87	2418	0.57
miRanda	All	93	6641	
	SVM	89	4301	0.82
	PITA	88	4942	0.69
	TargetScan	82	3072	0.8
	miRanda	93	6641	0.61
	PITA_top	31	801	0.61
	TargetScan_consv	36	819	0.64
	mirTarget2	41	602	0.67
	PicTar	25	554	0.6
PicTar	All	124	3826	
	SVM	119	3683	0.69
	PITA	122	3420	0.61
	TargetScan	105	3003	0.63
	miRanda	25	554	0.53
	PITA_top	92	2152	0.63
	TargetScan_consv	87	2406	0.61
	mirTarget2	77	1408	0.67
	PicTar	124	3826	0.65

[a]Positive and Negative show the total number of positive (down-regulated) and negative (unaffected) genes present within the six benchmarks (Method “All”) and among the predictions of each method on the six benchmarks. [b]ROC is the method’s ROC score on the specific benchmark.

Table S6. mRNA/siRNA sequences from five microarray experiments. Jackson (GSE5814), Lim (GSE2075), Grimson (GSE8501), and Birmingham (E-MEXP-668) were used for training, and Linsley (GSE6838) was used for the independent test. Two datasets, Selbach and Baek, from mass spectrometry experiments were used as both training datasets (for the proteomics-based classifiers) and independent test sets (for the mRNA-based classifiers).

Accession	miRNA/siRNA name	Sequence	Strand	miRNA/siRNA
GSE5814	MAPK14-pos1 mismatch	CCUACAGAGAACUGCGGUA	sense	siRNA
GSE5814	MAPK14-pos2 mismatch	CCUACAGAGAACUGCGGAU	sense	siRNA
GSE5814	MAPK14-pos8 mismatch	CCUACAGAGAAAUGCGGUU	sense	siRNA
GSE5814	MAPK14-pos7 mismatch	CCUACAGAGAACGGCGGUU	sense	siRNA
GSE5814	MAPK14-pos6 mismatch	CCUACAGAGAACUACGGUU	sense	siRNA
GSE5814	MAPK14-pos4 mismatch	CCUACAGAGAACUGCAGUU	sense	siRNA
GSE5814	MAPK14-pos3 mismatch	CCUACAGAGAACUGCGAUU	sense	siRNA
GSE5814	MAPK14-193[a]	CCUACAGAGAACUGCGGUU	sense	siRNA
GSE5814	PLK1 1319	CACGCCTCATCCTCTACAA	sense	siRNA
GSE5814	PLK1 772	GAGACCTACCTCCGGATCA	sense	siRNA
GSE5814	MAPK14-pos5 mismatch	CCUACAGAGAACUGAGGUU	sense	siRNA
GSE5814	PIK3CB-6338	GUGACAACAUCAUGGUCAA	sense	siRNA
GSE5814	PIK3CB-6340	CUCCUAAUAUGAAUCCUAU	sense	siRNA
GSE2075	miR-1	UGGAAUGUAAAGAAGUAUGUAA	antisense	miRNA
GSE2075	miR-124	UAAGGCACGCGGUGAAUGCCA	antisense	miRNA
GSE2075	miR-124mut5-6	UAAGCGACGCGGUGAAUGCCA	antisense	siRNA
GSE2075	miR-373	GAAGUGCUUCGAUUUUGGGGUGU	antisense	miRNA
GSE6838	hsa-miR-16	UAGCAGCACGUAAAUAUUGGCG	antisense	miRNA
GSE6838	hsa-miR-15a	UAGCAGCACAUAAUGGUUUGUG	antisense	miRNA
GSE6838	hsa_miR_106b	UAAAGUGCUGACAGUGCAGAU	antisense	miRNA
GSE6838	hsa_miR_103	AGCAGCAUUGUACAGGGCUAUGA	antisense	miRNA
GSE6838	hsa-miR-192	CUGACCUAUGAAUUGACAGCC	antisense	miRNA
GSE6838	hsa-miR-215	AUGACCUAUGAAUUGACAGAC	antisense	miRNA
GSE6838	hsa-miR-17-5p	CAAAGUGCUUACAGUGCAGGUAGU	antisense	miRNA
GSE6838	hsa-miR-20	UAAAGUGCUUAUAGUGCAGGUA	antisense	miRNA
GSE6838	hsa-let-7c	UGAGGUAGUAGGUUGUAUGGUU	antisense	miRNA
GSE8501	hsa-miR-7	UGGAAGACUAGUGAUUUUGUUG	antisense	miRNA
GSE8501	hsa-miR-9	UCUUUGGUUAUCUAGCUGUAUGA	antisense	miRNA
GSE8501	hsa-miR-122a	UGGAGUGUGACAAUGGUGUUUGU	antisense	miRNA
GSE8501	hsa-miR-128a	UCACAGUGAACCGGUCUCUUUU	antisense	miRNA
GSE8501	hsa-miR-132	UAACAGUCUACAGCCAUGGUCG	antisense	miRNA
GSE8501	hsa-miR-133a	UUGGUCCCCUUCAACCAGCUGU	antisense	miRNA
GSE8501	hsa-miR-142-3p	UGUAGUGUUUCCUACUUUAUGGA	antisense	miRNA
GSE8501	hsa-miR-148a	UCAGUGCACUACAGAACUUUGU	antisense	miRNA
GSE8501	hsa-miR-181a	AACAUUCAACGCUGUCGGUGAGU	antisense	miRNA
E-MEXP-668	C52	CAGGGCGGAGACTTCACCA	sense	siRNA
E-MEXP-668	G4	TGGTTTACATGTTCCAATA	sense	siRNA
E-MEXP-668	C4	GGAAAGACTGTTCCAAAAA	sense	siRNA
E-MEXP-668	G41	GTATGACAACAGCCTCAAG	sense	siRNA
E-MEXP-668	M3	GAGGTTCTCTGGATCAAGT	sense	siRNA
E-MEXP-668	M1	GCACATGGATGGAGGTTCT	sense	siRNA
E-MEXP-668	M2	GCAGAGAGAGCAGATTTGA	sense	siRNA
E-MEXP-668	C14	GGCCTTAGCTACAGGAGAG	sense	siRNA
E-MEXP-668	C1	GAAAGAGCATCTACGGTGA	sense	siRNA
E-MEXP-668	C2	GAAAGGATTTGGCTACAAA	sense	siRNA
E-MEXP-668	C3	ACAGCAAATTCCATCGTGT	sense	siRNA
E-MEXP-668	M4	GAGCAGATTTGAAGCAACT	sense	siRNA
Selbach	hsa-miR-30a	UGUAAACAUCCUCGACUGGAAG	antisense	miRNA
Selbach	hsa-miR-16	UAGCAGCACGUAAAUAUUGGCG	antisense	miRNA
Selbach	hsa-miR-155	UUAAUGCUAAUCGUGAUAGGGGU	antisense	miRNA
Selbach	hsa-miR-1	UGGAAUGUAAAGAAGUAUGUAU	antisense	miRNA
Selbach	hsa-let-7b	UGAGGUAGUAGGUUGUGUGGUU	antisense	miRNA
Baek	hsa-mir-181a	AACAUUCAACGCUGUCGGUGAGU	antisense	miRNA
Baek	hsa-mir-124	UAAGGCACGCGGUGAAUGCC	antisense	miRNA
Baek	hsa-mir-1	UGGAAUGUAAAGAAGUAUGUAU	antisense	miRNA

[a]MAPK14-193 has three different sample groups; (i) GSM133702 and GSM134468, (ii) GSM134488, and (iii) GSM134491. We assigned three different unique IDs to these groups, therefore they were treated as different sequences internally in our model.

Table S7. Parameters used for the Needle software. The scoring matrix was generated based on the matrix used in the miRanda algorithm (Enright, John et al. 2003).

<table>
<tr><td>Parameter</td><td colspan="5">Value</td></tr>
<tr><td>Gap open (-gapopen)</td><td colspan="5">10.0</td></tr>
<tr><td>Gap extend (-gapextend)</td><td colspan="5">7.0</td></tr>
<tr><td rowspan="5">Scoring matrix (-datafile)</td><td></td><td>A</td><td>U</td><td>G</td><td>C</td></tr>
<tr><td>A</td><td>-3</td><td>5</td><td>-3</td><td>-3</td></tr>
<tr><td>U</td><td>5</td><td>-3</td><td>2</td><td>-3</td></tr>
<tr><td>G</td><td>-3</td><td>2</td><td>-3</td><td>5</td></tr>
<tr><td>C</td><td>-3</td><td>-3</td><td>5</td><td>-3</td></tr>
</table>

Table S8. Benchmarks on the Selbach dataset

Benchmark	Method	# of targets[a]		ROC[b]
		Positive	Negative	
All genes	All	2311	12890	
	SVM	1493	5936	0.64
	PITA	1230	4276	0.61
	TargetScan	688	1220	0.61
	miRanda	183	493	0.52
	PITA_top	266	274	0.55
	TargetScan_consv	305	309	0.55
	mirTarget2	205	155	0.54
	PicTar	185	175	0.53
ROC_{10*n}	All	2311	50	
	SVM	95	50	0.0253
	PITA	17	50	0.0042
	TargetScan	84	50	0.0212
	miRanda	28	50	0.0079
	PITA_top	57	50	0.0138
	TargetScan_consv	82	50	0.0213
	mirTarget2	83	50	0.0231
	PicTar	81	50	0.021
7mer + Conservation	All	576	1005	
	SVM	560	800	0.71
	PITA	554	835	0.61
	TargetScan	495	616	0.69
	miRanda	183	493	0.42
	PITA_top	266	274	0.6
	TargetScan_consv	305	309	0.63
	mirTarget2	205	155	0.6
	PicTar	185	175	0.58
TargetScan	All	306	313	
	SVM	305	298	0.63
	PITA	302	310	0.52
	TargetScan	305	309	0.63
	miRanda	76	41	0.56
	PITA_top	187	172	0.55
	TargetScan_consv	305	309	0.63
	mirTarget2	133	84	0.59
	PicTar	139	125	0.55
miRanda	All	183	493	
	SVM	169	305	0.76
	PITA	166	328	0.66
	TargetScan	131	152	0.74
	miRanda	183	493	0.54
	PITA_top	68	41	0.64
	TargetScan_consv	76	39	0.67
	mirTarget2	58	28	0.63
	PicTar	54	29	0.62
PicTar	All	185	175	
	SVM	185	172	0.61
	PITA	184	172	0.54
	TargetScan	168	163	0.62
	miRanda	54	29	0.56
	PITA_top	126	94	0.58
	TargetScan_consv	139	124	0.6
	mirTarget2	86	47	0.6
	PicTar	185	175	0.62

[a]Positive and Negative show the total number of positive (down-regulated) and negative (unaffected) genes present within the six benchmarks (Method "All") and among the predictions of each method on the six benchmarks. [b]ROC is the method's ROC score on the specific benchmark.

Table S9. Benchmarks on the Baek dataset

Benchmark	Method	# of targets[a]		ROC[b]
		Positive	Negative	
All genes	All	1301	4257	
	SVM	725	2005	0.56
	PITA	605	1481	0.56
	TargetScan	319	557	0.56
	miRanda	81	216	0.51
	PITA_top	103	143	0.52
	TargetScan_consv	142	172	0.53
	mirTarget2	70	92	0.52
	PicTar	73	102	0.52
ROC_{10*n}	All	1301	30	
	SVM	39	30	0.0193
	PITA	15	30	0.0046
	TargetScan	28	30	0.0157
	miRanda	17	30	0.0081
	PITA_top	31	30	0.0148
	TargetScan_consv	32	30	0.0174
	mirTarget2	23	30	0.0086
	PicTar	29	30	0.0131
7mer + Conservation	All	258	486	
	SVM	235	407	0.59
	PITA	234	389	0.6
	TargetScan	217	322	0.62
	miRanda	81	216	0.44
	PITA_top	103	143	0.56
	TargetScan_consv	142	172	0.61
	mirTarget2	70	92	0.54
	PicTar	73	102	0.54
TargetScan	All	142	176	
	SVM	136	169	0.57
	PITA	135	164	0.57
	TargetScan	142	172	0.57
	miRanda	28	32	0.5
	PITA_top	86	98	0.56
	TargetScan_consv	142	172	0.57
	mirTarget2	48	54	0.52
	PicTar	51	58	0.52
miRanda	All	81	216	
	SVM	65	144	0.64
	PITA	70	142	0.62
	TargetScan	51	83	0.62
	miRanda	81	216	0.5
	PITA_top	21	32	0.56
	TargetScan_consv	28	31	0.59
	mirTarget2	19	25	0.55
	PicTar	16	16	0.57
PicTar	All	73	102	
	SVM	73	100	0.54
	PITA	71	91	0.62
	TargetScan	65	85	0.63
	miRanda	16	16	0.53
	PITA_top	42	48	0.6
	TargetScan_consv	51	57	0.63
	mirTarget2	27	26	0.56
	PicTar	73	102	0.59

[a]Positive and Negative show the total number of positive (down-regulated) and negative (unaffected) genes present within the six benchmarks (Method "All") and among the predictions of each method on the six benchmarks. [b]ROC is the method's ROC score on the specific benchmark.

Table S10. Benchmarks on the Linsley dataset – Positive records defined by log ratio -0.1

Benchmark	Method	# of targets[a]		ROC[b]
		Positive	Negative	
All genes	All	851	158190	
	SVM	692	68909	0.79
	PITA	655	55837	0.74
	TargetScan	474	21154	0.73
	miRanda	110	6624	0.54
	PITA_top	180	4834	0.59
	TargetScan_consv	221	5587	0.61
	mirTarget2	231	3346	0.62
	PicTar	159	3791	0.58
ROC_{10*n}	All	851	90	
	SVM	20	90	0.0133
	PITA	1	90	0.0014
	TargetScan	13	90	0.0147
	miRanda	13	90	0.0093
	PITA_top	4	90	0.0039
	TargetScan_consv	14	90	0.0142
	mirTarget2	22	90	0.0186
	PicTar	19	90	0.0106
7mer + Conservation	All	425	15751	
	SVM	409	12661	0.73
	PITA	404	13165	0.60
	TargetScan	368	10657	0.70
	miRanda	110	6624	0.43
	PITA_top	180	4834	0.57
	TargetScan_consv	221	5587	0.61
	mirTarget2	231	3346	0.67
	PicTar	159	3791	0.58
TargetScan	All	221	5611	
	SVM	215	4975	0.67
	PITA	219	5395	0.55
	TargetScan	221	5587	0.66
	miRanda	48	815	0.54
	PITA_top	151	3495	0.55
	TargetScan_consv	221	5587	0.66
	mirTarget2	134	1901	0.65
	PicTar	116	2389	0.58
miRanda	All	110	6624	
	SVM	104	4286	0.81
	PITA	105	4925	0.71
	TargetScan	97	3057	0.81
	miRanda	110	6624	0.62
	PITA_top	39	793	0.62
	TargetScan_consv	48	807	0.67
	mirTarget2	49	594	0.68
	PicTar	30	549	0.60
PicTar	All	159	3791	
	SVM	154	3648	0.68
	PITA	154	3388	0.59
	TargetScan	135	2973	0.63
	miRanda	30	549	0.52
	PITA_top	113	2131	0.60
	TargetScan_consv	116	2377	0.61
	mirTarget2	94	1391	0.64
	PicTar	159	3791	0.63

[a]Positive and Negative show the total number of positive (down-regulated) and negative (unaffected) genes present within the six benchmarks (Method “All”) and among the predictions of each method on the six benchmarks. [b]ROC is the method’s ROC score on the specific benchmark.

Table S11. Benchmarks on the Linsley dataset – Positive records defined by log ratio -0.3

Benchmark	Method	# of targets[a]		ROC[b]
		Positive	Negative	
All genes	All	128	158913	
	SVM	111	69490	0.83
	PITA	109	56383	0.80
	TargetScan	84	21544	0.78
	miRanda	19	6715	0.55
	PITA_top	21	4993	0.57
	TargetScan_consv	21	5787	0.57
	mirTarget2	38	3539	0.64
	PicTar	20	3930	0.57
ROC_{10*n}	All	128	90	
	SVM	1	90	0.0148
	PITA	0	90	0.0078
	TargetScan	1	90	0.0148
	miRanda	3	90	0.0237
	PITA_top	1	90	0.0109
	TargetScan_consv	0	90	0.0078
	mirTarget2	1	90	0.0134
	PicTar	2	90	0.0151
7mer + Conservation	All	69	16107	
	SVM	68	13002	0.74
	PITA	64	13505	0.61
	TargetScan	55	10970	0.63
	miRanda	19	6715	0.44
	PITA_top	21	4993	0.51
	TargetScan_consv	21	5787	0.49
	mirTarget2	38	3539	0.68
	PicTar	20	3930	0.53
TargetScan	All	21	5811	
	SVM	21	5169	0.76
	PITA	21	5593	0.57
	TargetScan	21	5787	0.70
	miRanda	8	855	0.62
	PITA_top	16	3630	0.61
	TargetScan_consv	21	5787	0.70
	mirTarget2	13	2022	0.67
	PicTar	12	2493	0.61
miRanda	All	19	6715	
	SVM	19	4371	0.87
	PITA	18	5012	0.73
	TargetScan	19	3135	0.88
	miRanda	19	6715	0.59
	PITA_top	8	824	0.65
	TargetScan_consv	8	847	0.66
	mirTarget2	9	634	0.69
	PicTar	4	575	0.57
PicTar	All	20	3930	
	SVM	20	3782	0.72
	PITA	20	3522	0.71
	TargetScan	17	3091	0.65
	miRanda	4	575	0.54
	PITA_top	14	2230	0.62
	TargetScan_consv	12	2481	0.55
	mirTarget2	12	1473	0.64
	PicTar	20	3930	0.65

[a]Positive and Negative show the total number of positive (down-regulated) and negative (unaffected) genes present within the six benchmarks (Method “All”) and among the predictions of each method on the six benchmarks. [b]ROC is the method’s ROC score on the specific benchmark.

Table S12. Benchmarks on the Selbach dataset – Positive records defined by log ratio -0.1

Benchmark	Method	# of targets[a]		ROC[b]
		Positive	Negative	
All genes	All	4221	10980	
	SVM	2451	4978	0.59
	PITA	1946	3560	0.58
	TargetScan	929	979	0.57
	miRanda	279	397	0.52
	PITA_top	331	209	0.53
	TargetScan_consv	379	235	0.53
	mirTarget2	248	112	0.52
	PicTar	237	123	0.52
ROC_{10*n}	All	4221	50	
	SVM	126	50	0.0168
	PITA	35	50	0.0033
	TargetScan	114	50	0.0144
	miRanda	41	50	0.0061
	PITA_top	105	50	0.0135
	TargetScan_consv	112	50	0.015
	mirTarget2	98	50	0.0159
	PicTar	152	50	0.0196
7mer + Conservation	All	793	788	
	SVM	738	622	0.66
	PITA	734	655	0.58
	TargetScan	629	482	0.65
	miRanda	279	397	0.43
	PITA_top	331	209	0.58
	TargetScan_consv	379	235	0.6
	mirTarget2	248	112	0.58
	PicTar	237	123	0.58
TargetScan	All	382	237	
	SVM	379	224	0.62
	PITA	377	235	0.52
	TargetScan	379	235	0.6
	miRanda	86	31	0.55
	PITA_top	230	129	0.55
	TargetScan_consv	379	235	0.6
	mirTarget2	157	60	0.58
	PicTar	177	87	0.57
miRanda	All	279	397	
	SVM	227	247	0.67
	PITA	225	269	0.58
	TargetScan	160	123	0.66
	miRanda	279	397	0.53
	PITA_top	78	31	0.6
	TargetScan_consv	85	30	0.61
	mirTarget2	64	22	0.58
	PicTar	62	21	0.58
PicTar	All	237	123	
	SVM	236	121	0.59
	PITA	235	121	0.57
	TargetScan	216	115	0.59
	miRanda	62	21	0.55
	PITA_top	154	66	0.57
	TargetScan_consv	176	87	0.58
	mirTarget2	101	32	0.58
	PicTar	237	123	0.64

[a]Positive and Negative show the total number of positive (down-regulated) and negative (unaffected) genes present within the six benchmarks (Method “All”) and among the predictions of each method on the six benchmarks. [b]ROC is the method’s ROC score on the specific benchmark.

Table S13. Benchmarks on the Selbach dataset – Positive records defined by log ratio -0.3

Benchmark	Method	# of targets[a]		ROC[b]
		Positive	Negative	
All genes	All	1311	13890	
	SVM	933	6496	0.68
	PITA	805	4701	0.65
	TargetScan	504	1404	0.65
	miRanda	131	545	0.53
	PITA_top	200	340	0.56
	TargetScan_consv	231	383	0.58
	mirTarget2	168	192	0.55
	PicTar	145	215	0.55
ROC_{10*n}	All	1311	50	
	SVM	75	50	0.0369
	PITA	12	50	0.0045
	TargetScan	67	50	0.0322
	miRanda	21	50	0.0111
	PITA_top	33	50	0.0142
	TargetScan_consv	64	50	0.0306
	mirTarget2	58	50	0.0264
	PicTar	55	50	0.0258
7mer + Conservation	All	423	1158	
	SVM	414	946	0.72
	PITA	410	979	0.61
	TargetScan	372	739	0.71
	miRanda	131	545	0.42
	PITA_top	200	340	0.6
	TargetScan_consv	231	383	0.64
	mirTarget2	168	192	0.61
	PicTar	145	215	0.59
TargetScan	All	231	388	
	SVM	230	373	0.65
	PITA	229	383	0.52
	TargetScan	231	383	0.67
	miRanda	61	56	0.56
	PITA_top	144	215	0.55
	TargetScan_consv	231	383	0.67
	mirTarget2	109	108	0.6
	PicTar	110	154	0.57
miRanda	All	131	545	
	SVM	123	351	0.78
	PITA	120	374	0.63
	TargetScan	101	182	0.77
	miRanda	131	545	0.53
	PITA_top	50	59	0.63
	TargetScan_consv	61	54	0.69
	mirTarget2	50	36	0.65
	PicTar	42	41	0.62
PicTar	All	145	215	
	SVM	145	212	0.64
	PITA	144	212	0.52
	TargetScan	135	196	0.66
	miRanda	42	41	0.55
	PITA_top	100	120	0.56
	TargetScan_consv	110	153	0.63
	mirTarget2	73	60	0.62
	PicTar	145	215	0.6

[a]Positive and Negative show the total number of positive (down-regulated) and negative (unaffected) genes present within the six benchmarks (Method “All”) and among the predictions of each method on the six benchmarks. [b]ROC is the method’s ROC score on the specific benchmark.

Table S14. Benchmarks on the Baek dataset – Positive records defined by log ratio -0.1

Benchmark	Method	# of targets[a]		ROC[b]
		Positive	Negative	
All genes	All	1992	3566	
	SVM	1062	1668	0.55
	PITA	861	1225	0.55
	TargetScan	446	430	0.55
	miRanda	113	184	0.5
	PITA_top	140	106	0.52
	TargetScan_consv	191	123	0.53
	mirTarget2	99	63	0.52
	PicTar	100	75	0.51
ROC_{10*n}	All	1992	30	
	SVM	63	30	0.0196
	PITA	30	30	0.0055
	TargetScan	56	30	0.016
	miRanda	21	30	0.0069
	PITA_top	51	30	0.0155
	TargetScan_consv	53	30	0.017
	mirTarget2	57	30	0.014
	PicTar	54	30	0.0138
7mer + Conservation	All	364	380	
	SVM	331	311	0.6
	PITA	322	301	0.57
	TargetScan	300	239	0.63
	miRanda	113	184	0.41
	PITA_top	140	106	0.56
	TargetScan_consv	191	123	0.61
	mirTarget2	99	63	0.55
	PicTar	100	75	0.54
TargetScan	All	192	126	
	SVM	184	121	0.56
	PITA	181	118	0.51
	TargetScan	191	123	0.58
	miRanda	36	24	0.5
	PITA_top	115	69	0.55
	TargetScan_consv	191	123	0.58
	mirTarget2	68	34	0.55
	PicTar	63	46	0.49
miRanda	All	113	184	
	SVM	89	120	0.64
	PITA	91	121	0.6
	TargetScan	67	67	0.61
	miRanda	113	184	0.49
	PITA_top	27	26	0.56
	TargetScan_consv	36	23	0.59
	mirTarget2	27	17	0.57
	PicTar	22	10	0.57
PicTar	All	100	75	
	SVM	100	73	0.59
	PITA	97	65	0.65
	TargetScan	89	61	0.64
	miRanda	22	10	0.54
	PITA_top	56	34	0.6
	TargetScan_consv	63	45	0.59
	mirTarget2	37	16	0.59
	PicTar	100	75	0.58

[a]Positive and Negative show the total number of positive (down-regulated) and negative (unaffected) genes present within the six benchmarks (Method “All”) and among the predictions of each method on the six benchmarks. [b]ROC is the method’s ROC score on the specific benchmark.

Table S15. Benchmarks on the Baek dataset – Positive records defined by log ratio -0.3

Benchmark	Method	# of targets[a]		ROC[b]
		Positive	Negative	
All genes	All	837	4721	
	SVM	492	2238	0.58
	PITA	416	1670	0.57
	TargetScan	228	648	0.57
	miRanda	50	247	0.50
	PITA_top	83	163	0.53
	TargetScan_consv	113	201	0.55
	mirTarget2	59	103	0.52
	PicTar	53	122	0.52
ROC_{10*n}	All	837	30	
	SVM	29	30	0.0230
	PITA	8	30	0.0047
	TargetScan	24	30	0.0220
	miRanda	7	30	0.0080
	PITA_top	19	30	0.0143
	TargetScan_consv	30	30	0.0264
	mirTarget2	20	30	0.0107
	PicTar	16	30	0.0106
7mer + Conservation	All	184	560	
	SVM	171	471	0.62
	PITA	168	455	0.60
	TargetScan	163	376	0.66
	miRanda	50	247	0.42
	PITA_top	83	163	0.59
	TargetScan_consv	113	201	0.65
	mirTarget2	59	103	0.57
	PicTar	53	122	0.54
TargetScan	All	113	205	
	SVM	109	196	0.60
	PITA	106	193	0.56
	TargetScan	113	201	0.60
	miRanda	21	39	0.50
	PITA_top	72	112	0.57
	TargetScan_consv	113	201	0.60
	mirTarget2	44	58	0.55
	PicTar	39	70	0.50
miRanda	All	50	247	
	SVM	41	168	0.67
	PITA	44	168	0.61
	TargetScan	35	99	0.67
	miRanda	50	247	0.54
	PITA_top	14	39	0.56
	TargetScan_consv	21	38	0.63
	mirTarget2	15	29	0.57
	PicTar	10	22	0.56
PicTar	All	53	122	
	SVM	53	120	0.56
	PITA	52	110	0.59
	TargetScan	49	101	0.66
	miRanda	10	22	0.50
	PITA_top	34	56	0.63
	TargetScan_consv	39	69	0.67
	mirTarget2	22	31	0.59
	PicTar	53	122	0.57

[a]Positive and Negative show the total number of positive (down-regulated) and negative (unaffected) genes present within the six benchmarks (Method “All”) and among the predictions of each method on the six benchmarks. [b]ROC is the method’s ROC score on the specific benchmark.

Supplementary Figures

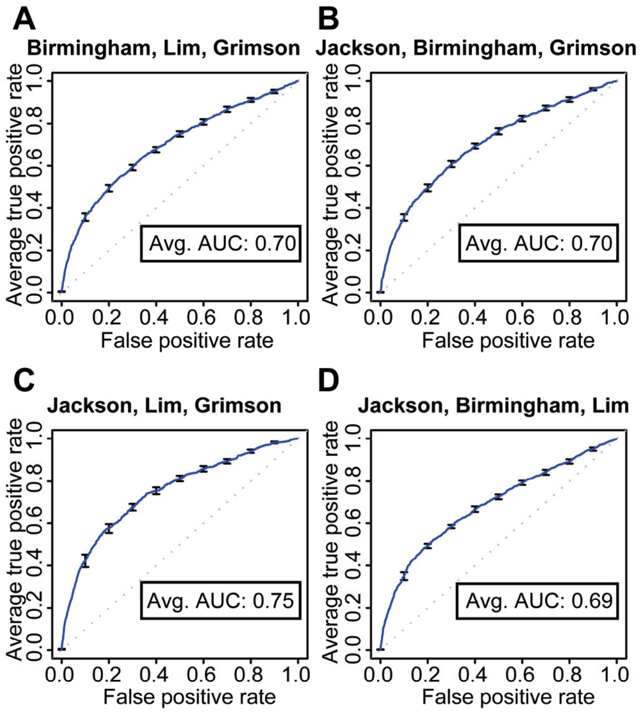

Figure S1. 10-fold cross-validation of target site level classifiers trained on three microarray datasets.

The ROC graphs show the classification performance of four classifiers trained with three microarray datasets at the target site level. The curve is the average ROC curve of the ten individual cross-validation test results; error bars show standard errors. Dotted lines illustrate random prediction. Avg. AUC is the average of the area under the curve (AUC; ROC score) of the ten individual cross-validation test results.

(A) Trained with the Birmingham, Lim and Grimson datasets.

(B) Trained with the Jackson, Birmingham and Grimson datasets

(C) Trained with the Jackson, Lim and Grimson datasets.

(D) Trained with the Jackson, Birmingham and Lim datasets.

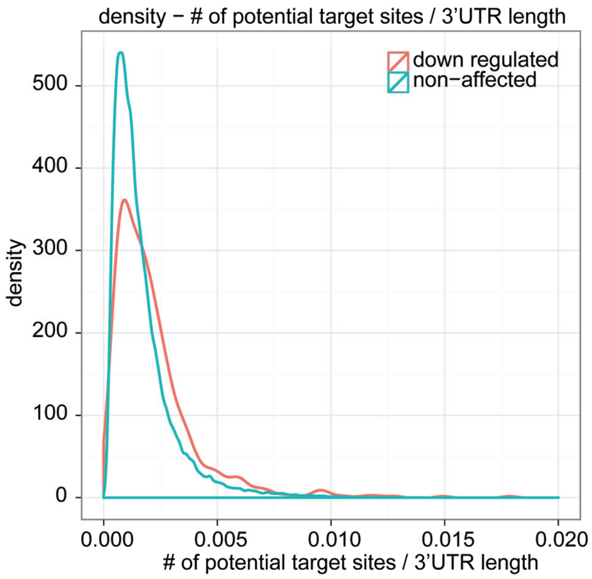

Figure S2. Density plot for the number of potential target sites divided by 3'UTR length. It shows the density distribution of the values calculated by the number of miRNA potential sites divided by the 3' UTR length. The red line indicates the distribution of down-regulated genes whereas the blue line is for the non-affected genes. The difference between two density distributions is very significant (Kolmogorov-Smirnov test, p-value: 3.862e-11).

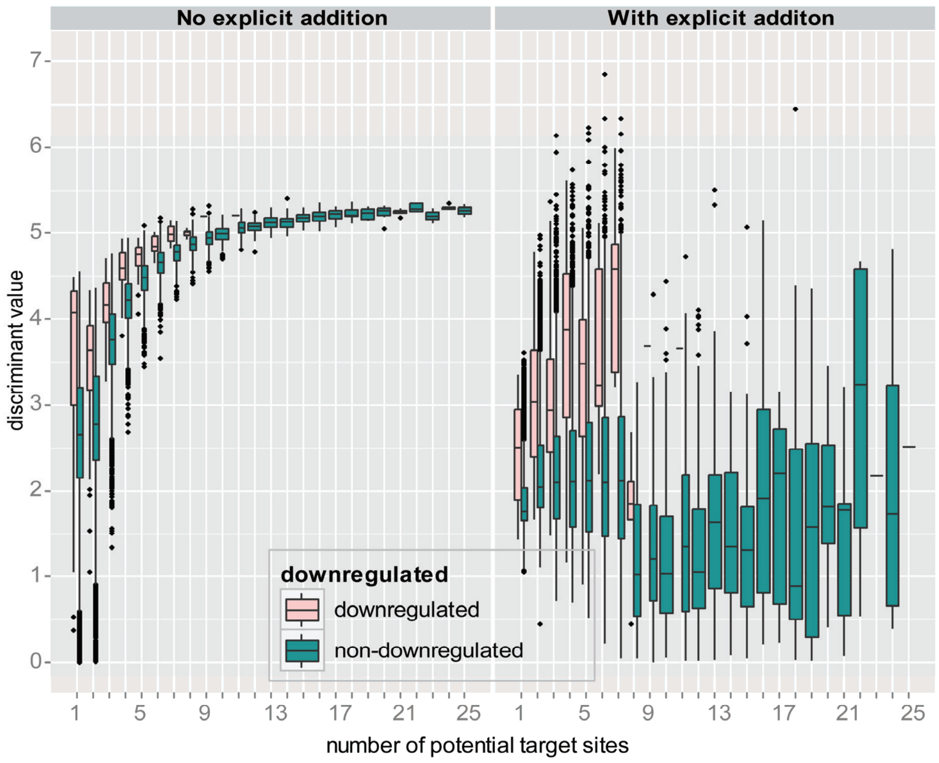

Figure S3. Comparison between two SVM trainings with or without explicit addition of negative records

Two box plots show the number of potential target sites vs. discriminant values from mRNA level training. The left panel shows the distributions of discriminant values by the number of potential target sites for the classifier trained without any explicit additional of negative records. The right panel shows the classifier trained with data enriched with 1000 randomly selected non-target genes with more than 7 target sites. Records with the number of target sites >25 and/or negative discriminant values were disregarded. Whereas the left box plot shows that discriminant values for negative (non-downregulated) genes generally increase with increasing number of potential target sites, the right box plot indicates that the discriminant values for negative genes are relatively stable.

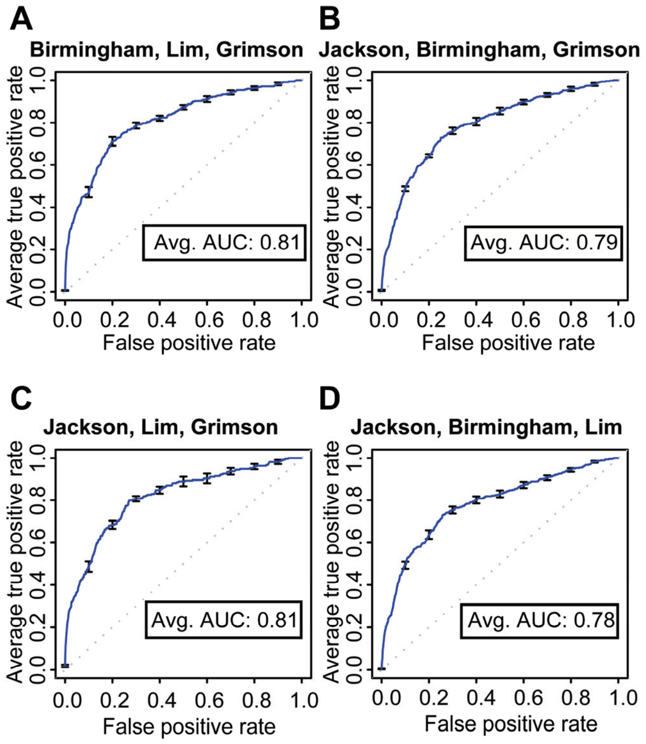

Figure S4. 10-fold cross-validation of mRNA level classifiers trained on three microarray datasets. The ROC graphs show the classification performance of four classifiers trained with three different combinations of microarray datasets at the mRNA level. The curve is the average ROC curve of the ten individual cross-validation test results; error bars show standard errors. Dotted lines illustrate a random prediction. Avg. AUC is the average of the area under the curve (AUC; ROC score) of the ten individual cross-validation test results.

(A) Trained with the Birmingham, Lim and Grimson datasets.

(B) Trained with the Jackson, Birmingham and Grimson datasets.

(C) Trained with the Jackson, Lim and Grimson datasets.

(D) Trained with the Jackson, Birmingham and Lim datasets.

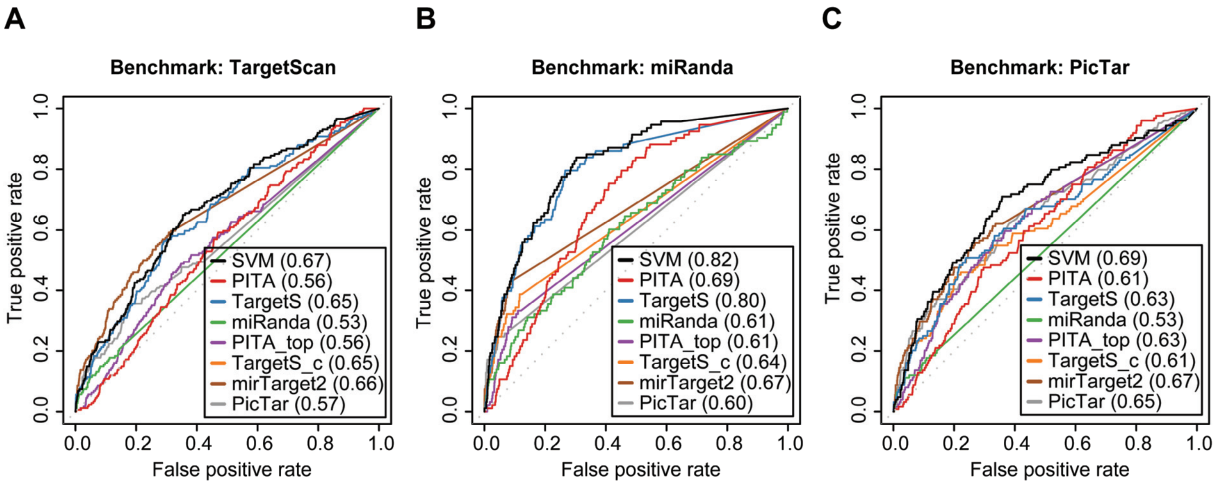

Figure S5. Three method-specific benchmarks of 8 different algorithms on the Linsley dataset. Receiver operating characteristic (ROC) graphs show the performances of 8 different target prediction algorithms; SVM, PITA All (PITA), PITA Top (PITA_top), TargetScan (TargetS), TargetScan with conserved genes (TargetS_c), MicroCosm miRanda (miRanda), mirTarget2, and PicTar, on the Linsley dataset. Dotted lines illustrate random prediction. The values of the area under the ROC curve (AUC) are shown in the legend box. The benchmarks used for the evaluation were (A) ROC with TargetScan dataset, (B) ROC with miRanda dataset, and (C) ROC with PicTar dataset.

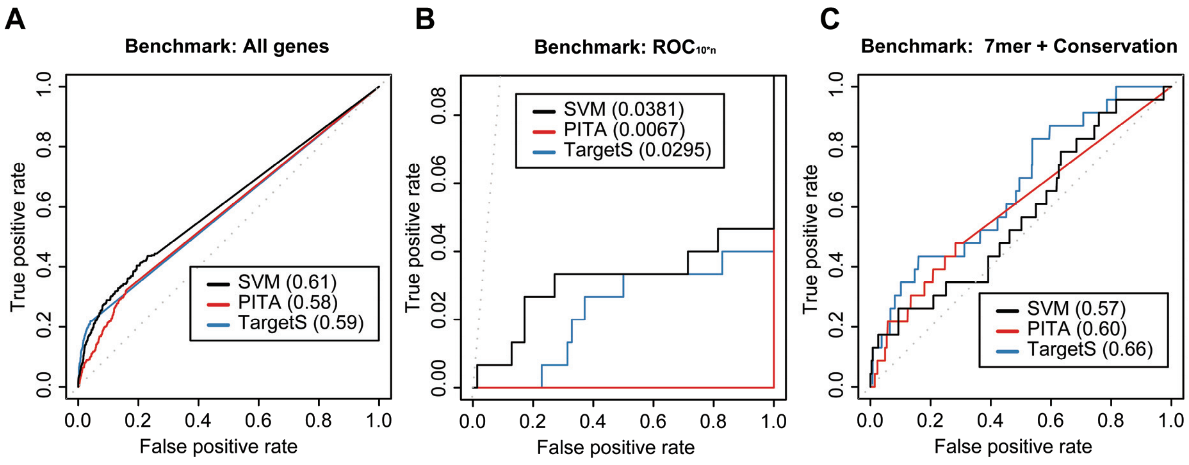

Figure S6. siRNA benchmarks on the Jackson dataset.

Receiver operating characteristic (ROC) graphs show the performance of 3 different target prediction algorithms – SVM not trained on Jackson dataset (SVM), PITA, and TargetScan (TargetS) – on the Jackson dataset. The SVM classifier was trained with the Lim, Birmingham and Grimson datasets. Dotted lines illustrate random prediction. The ROC scores are shown in the legend box. The benchmarks used for the evaluation were (A) ROC with All genes, (B) ROC_{10*n}, and (C) ROC with 7mer + Conservation.

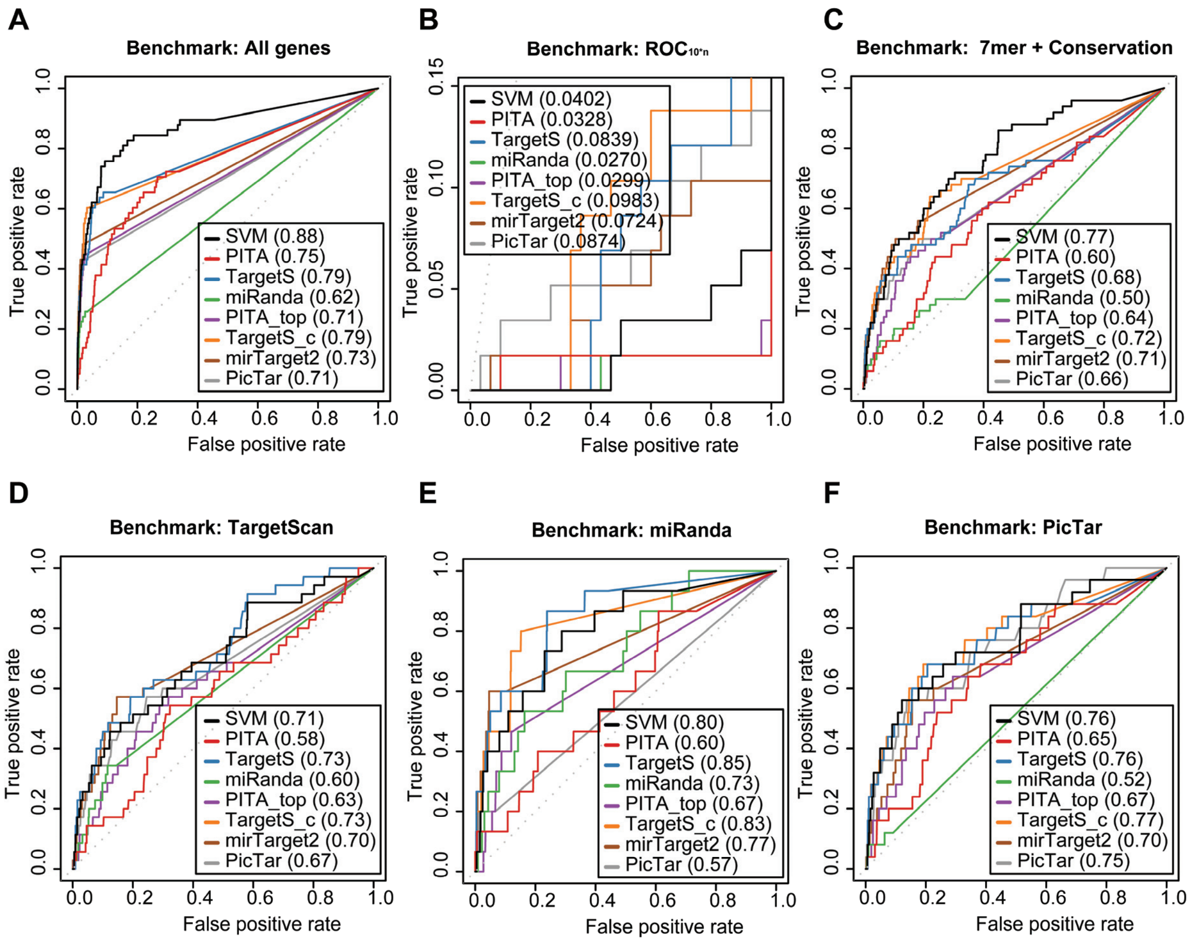

Figure S7. miRNA benchmarks on the Lim dataset.

Receiver operating characteristic (ROC) graphs show the performances of 8 different target prediction algorithms – SVM not trained on Lim dataset (SVM), PITA All (PITA), PITA Top (PITA_top), TargetScan (TargetS), TargetScan with conserved genes (TargetS_c), MicroCosm miRanda (miRanda), mirTarget2 and PicTar – on the Lim dataset. The SVM classifier was trained with the Jackon, Birmingham and Grimson datasets. Dotted lines illustrate random prediction. The values of the area under the ROC curve (AUC) are shown in the legend box. The benchmarks used for the evaluation were (A) ROC with TargetScan dataset, (B) ROC with miRanda dataset, (C) ROC with PicTar dataset, (D) ROC with TargetScan dataset, (E) ROC with miRanda dataset, and (F) ROC with PicTar dataset.

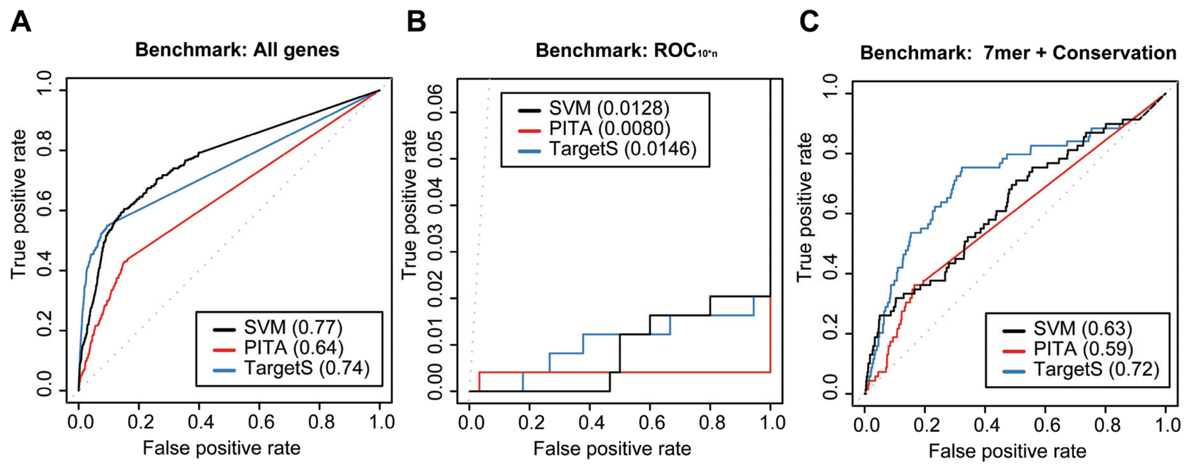

Figure S8. siRNA benchmarks on the Birmingham dataset.

Receiver operating characteristic (ROC) graphs show the performances of 3 different target prediction algorithms – SVM not trained on Birmingham dataset (SVM), PITA, and TargetScan (TargetS) – on the Birmingham dataset. The SVM classifier was trained with the Jackson, Lim and Grimon datasets. Dotted lines illustrate random prediction. The ROC scores are shown in the legend box. The benchmarks used for the evaluation were (A) ROC with All genes, (B) ROC_{10*n}, and (C) ROC with 7mer + Conservation.

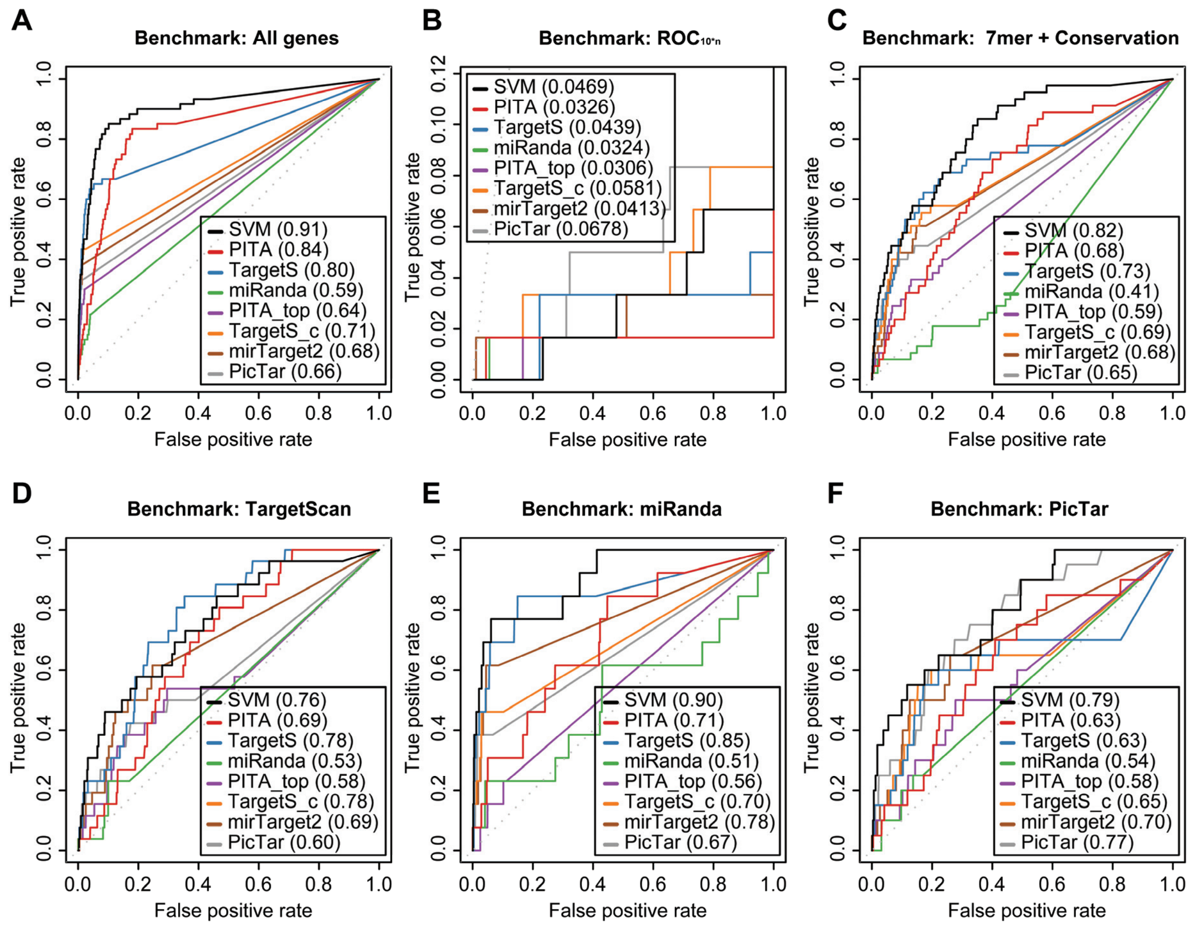

Figure S9. miRNA benchmarks on the Grimson dataset.

Receiver operating characteristic (ROC) graphs show the performances of 8 different target prediction algorithms – SVM not trained on Grimson dataset (SVM), PITA All (PITA), PITA Top (PITA_top), TargetScan (TargetS), TargetScan with conserved genes (TargetS_c), MicroCosm miRanda (miRanda), mirTarget2 and PicTar – on the Grimson dataset. The SVM classifier was trained with the Jackon, Lim, and Birmingham datasets. Dotted lines illustrate random prediction. The values of the area under the ROC curve (AUC) are shown in the legend box. The benchmarks used for the evaluation were (A) ROC with TargetScan dataset, (B) ROC with miRanda dataset, (C) ROC with PicTar dataset, (D) ROC with TargetScan dataset, (E) ROC with miRanda dataset, and (F) ROC with PicTar dataset.

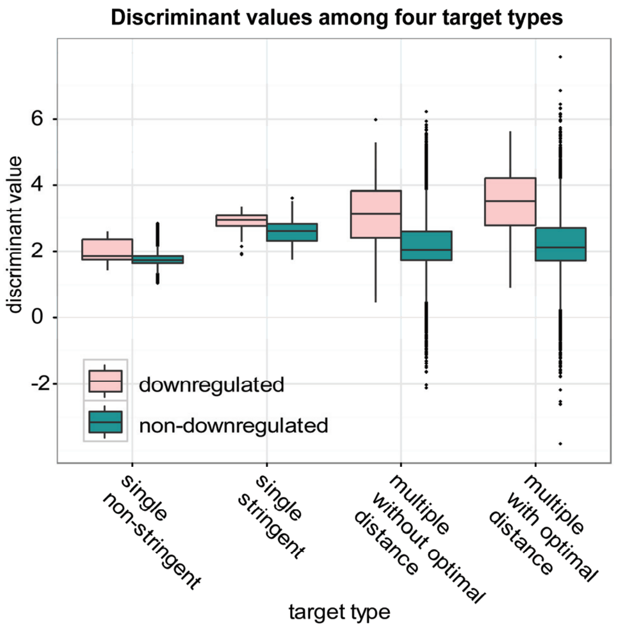

Figure S10. Targets with optimal distance sites show higher average discriminant value than other three target types.

The boxplot shows the comparison of mRNA level discriminant values among four different target types. The result shows our SVM approach gives scores as the preference: “multiple with optimal distance” > “multiple without optimal distance” > “single stringent” > “single non-stringent”.

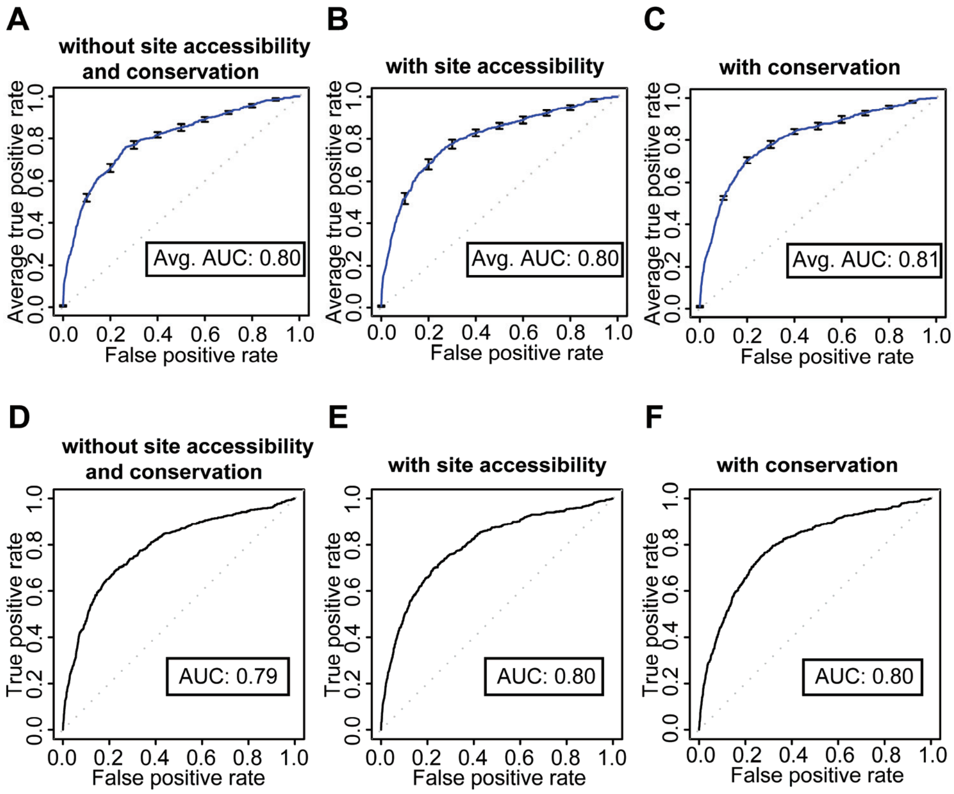

Figure S11. Adding site accessibility and sequence conservation features did not affect target prediction accuracy.

Receiver operating characteristic (ROC) graphs show the classification performance of three mRNA level classifiers. These classifiers had the same mRNA level features, but their discriminant values were generated by three different target site classifiers: (i) neither site accessibility nor sequence conservation used, (ii) with site accessibility and, (iii) with conservational information. Panels A-C show the three classifiers' 10-fold cross-validation performance. The curve is the average ROC curve of the ten individual cross-validation test results; error bars show standard errors; Avg. AUC is the average of the area under the curve (AUC; ROC score) of the ten individual cross-validation test results. Panels D-F show the three classifiers' performance on the independent (Linsley) dataset; ROC graphs were plotted by only considering the genes with prediction scores in the Linsley dataset. Dotted lines illustrate random prediction.

(A) 10-fold cross-validation of the classifier with neither site accessibility nor sequence conservation used

(B) 10-fold cross-validation of the classifier with site accessibility

(C) 10-fold cross-validation of the classifier with sequence conservation

(D) Classifier with neither site accessibility nor conservational information tested on the independent dataset

(E) Classifier with site accessibility tested on the independent dataset

(F) Classifier with sequence conservation tested on the independent dataset

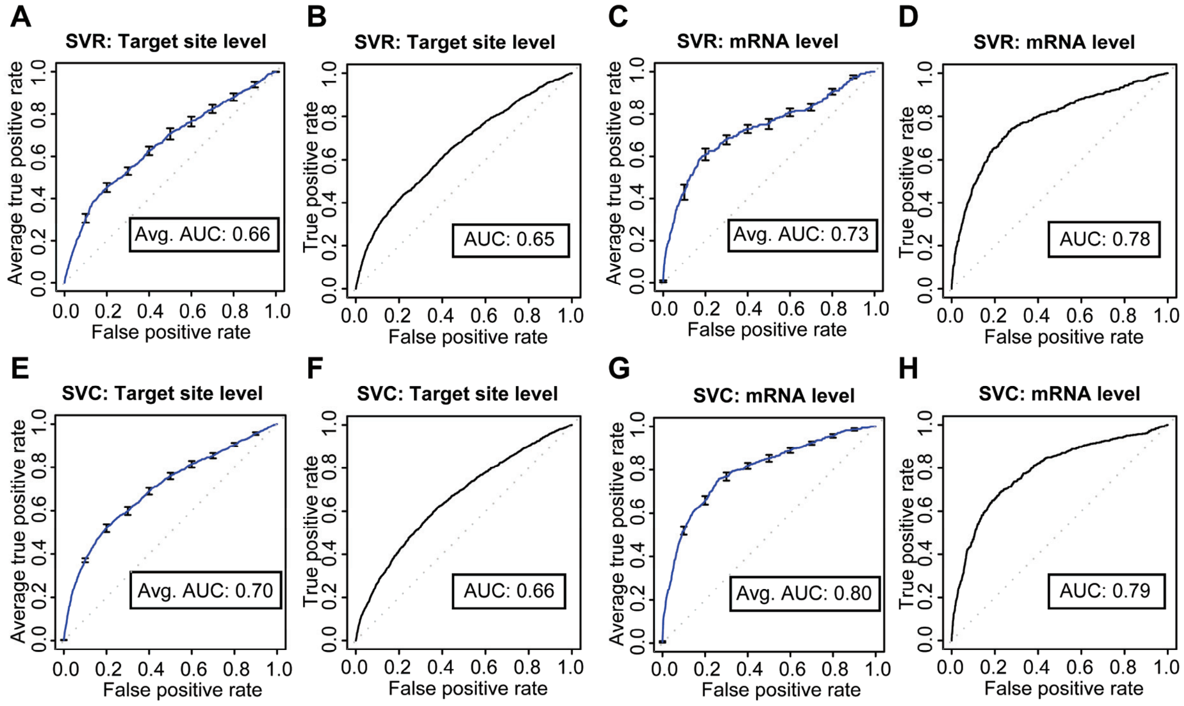

Figure S12. SVC performs better than SVR at both target site and mRNA levels.

The ROC graphs show the prediction performance of SVM regression (SVR) and SVM classification (SVC). Panels A, C, E, and G show the models' 10-fold cross-validation performance. The curve is the average ROC curve of the ten individual cross-validation test results; error bars show standard errors; Avg. AUC is the average of the area under the curve (AUC; ROC score) of the ten individual cross-validation test results. Panels B, D, F, and H show the models' performance on the independent (Linsley) dataset; ROC graphs were plotted by only considering the genes with SVC and SVR prediction scores in the Linsley dataset. Dotted lines illustrate random prediction. SVR and SVC show very similar performance, but SVC shows slightly better performance than SVR at both target site and mRNA levels.

(A) 10-fold cross-validation of SVR target site level

(B) Target site level SVR classifier tested on the independent dataset

(C) 10-fold cross-validation of SVR mRNA level

(D) mRNA level SVR classifier tested on the independent dataset

(E) 10-fold cross-validation of SVC target site level

(F) Target site level SVC classifier tested on the independent dataset

(G) 10-fold cross-validation of SVC mRNA level

(H) mRNA level SVC classifier tested on the independent dataset

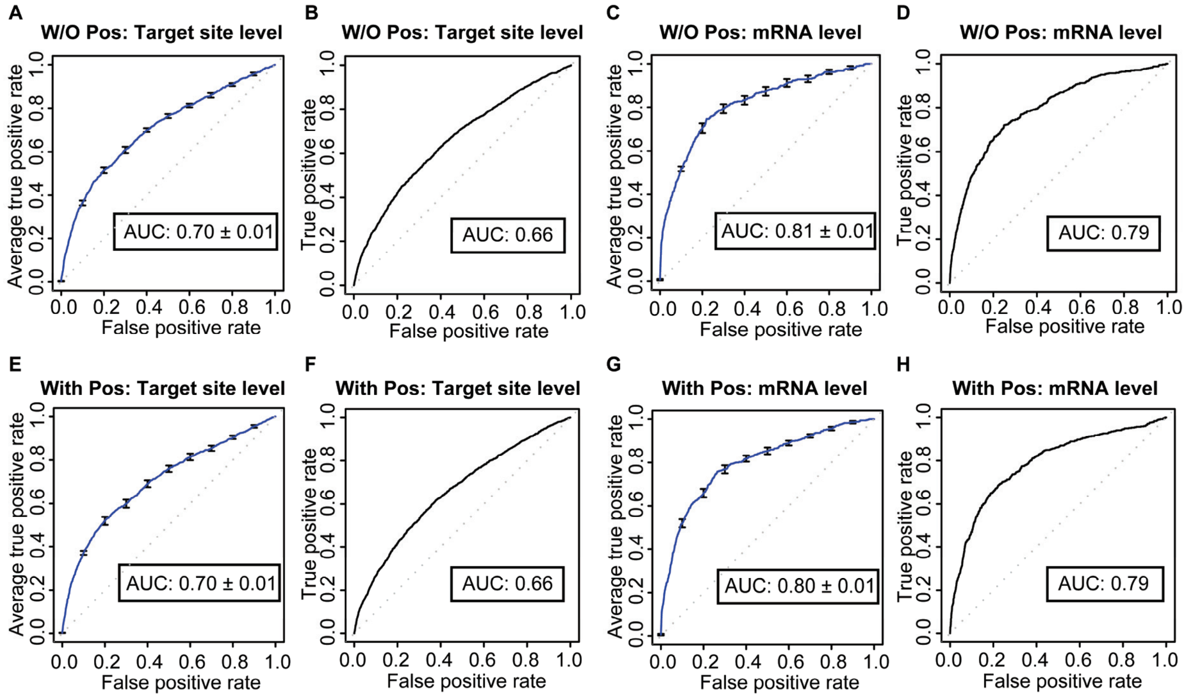

Figure S13. SVM performance is similar between the training set with up-regulated genes and the one without up-regulated genes.

The ROC graphs show the prediction performance of two SVM classifiers trained either with or without up-regulated genes. Panels A, C, E, and G show the models' 10-fold cross-validation performance. The curve is the average ROC curve of the ten individual cross-validation test results; error bars show standard errors; Avg. AUC is the average of the area under the curve (AUC; ROC score) of the ten individual cross-validation test results. Panels B, D, F, and H show the models' performance on the independent (Linsley) dataset; ROC graphs were plotted by only considering the genes with SVM prediction scores in the Linsley dataset. Dotted lines illustrate random prediction. Two classifiers show almost identical performances in terms of both ROC sores and ROC curves.

(A) 10-fold cross-validation of SVM without up-regulated genes at target site level

(B) Target site level SVM without up-regulated genes tested on the independent dataset

(C) 10-fold cross-validation of SVM without up-regulated genes at mRNA level

(D) mRNA level SVM without up-regulated genes tested on the independent dataset

(E) 10-fold cross-validation of SVM with up-regulated genes at target site level

(F) Target site level SVM with up-regulated genes tested on the independent dataset

(G) 10-fold cross-validation of SVM with up-regulated genes at mRNA level

(H) mRNA level SVM with up-regulated genes tested on the independent dataset

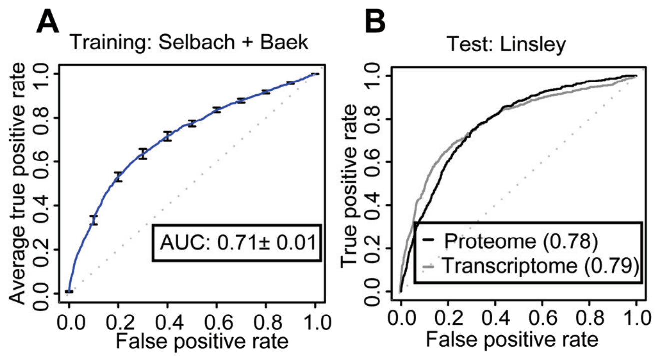

Figure S14. Two-step SVM retains the performance when trained with proteomics data
(A) SVM classifier was trained by the Selbach and Baek datasets. The definitions of the error bars, AUC, true positive and false positive rates were the same as described in Figure 2. (B) Both proteomics and transcriptomics classifiers were tested on the Linsley dataset. Proteomics data contains the Selbach and Baek datasets, whereas Transcriptomics contains the Jackson, Lim, Birmingham, and Grimson datasets. The ROC scores were shown in the legend box.

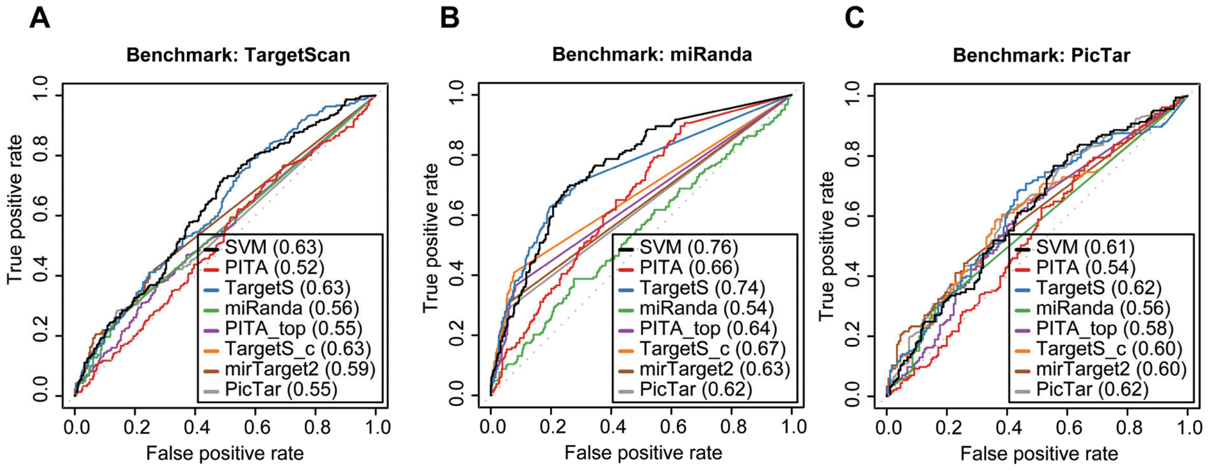

Figure S15. Benchmarks on the Selbach dataset

Receiver operating characteristic (ROC) graphs show the performances of 8 different target prediction algorithms – SVM, PITA All (PITA), PITA Top (PITA_top), TargetScan (TargetS), TargetScan with conserved genes (TargetS_c), MicroCosm miRanda (miRanda), mirTarget2 and PicTar – on the Selbach dataset. Dotted lines illustrate random prediction. The ROC scores are shown in the legend box. The benchmarks used for the evaluation were, (A) ROC with TargetScan dataset, (B) ROC with miRanda dataset, and (C) ROC with PicTar dataset.

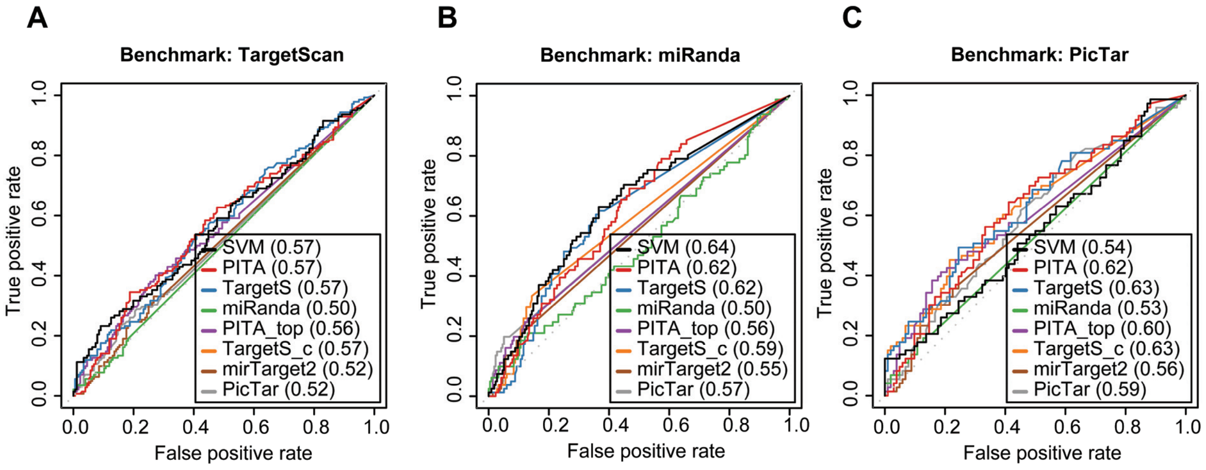

Figure S16. Benchmarks on the Baek dataset

Receiver operating characteristic (ROC) graphs show the performances of 8 different target prediction algorithms – SVM, PITA All (PITA), PITA Top (PITA_top), TargetScan (TargetS), TargetScan with conserved genes (TargetS_c), MicroCosm miRanda (miRanda), mirTarget2 and PicTar – on the Baek dataset. Dotted lines illustrate random prediction. The ROC scores are shown in the legend box. The benchmarks used for the evaluation were (A) ROC with TargetScan dataset, (B) ROC with miRanda dataset, and (C) ROC with PicTar dataset.

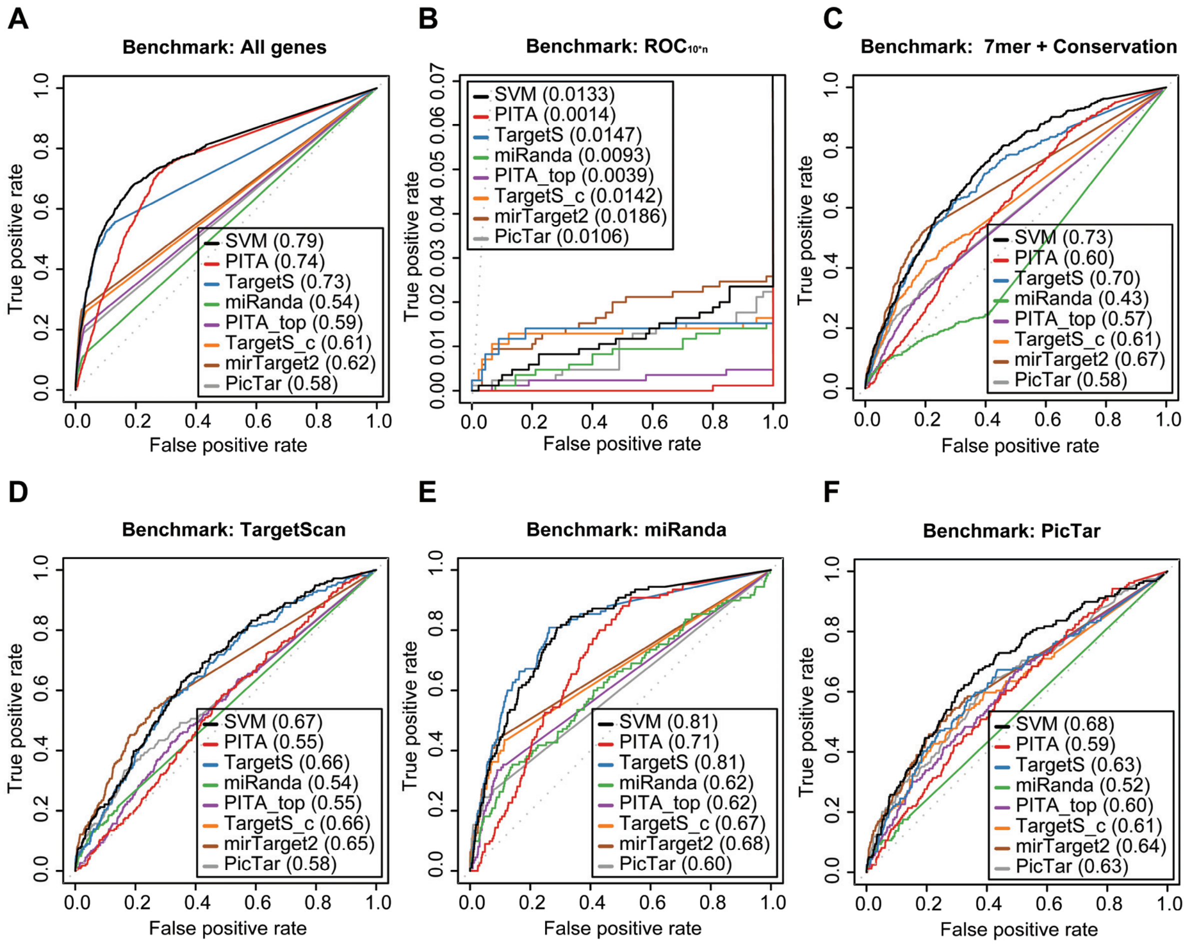

Figure S17. Benchmarks on the Linsley dataset with positive record threshold by log ratio -0.1

Receiver operating characteristic (ROC) graphs show the performances of 8 different target prediction algorithms – SVM, PITA All (PITA), PITA Top (PITA_top), TargetScan (TargetS), TargetScan with conserved genes (TargetS_c), MicroCosm miRanda (miRanda), mirTarget2 and PicTar – on the Linsley dataset. Dotted lines illustrate random prediction. The ROC scores are shown in the legend box. Positive records (down-regulated) were selected by log ratio value -0.1 instead of the default value -0.2. The benchmarks used for the evaluation were (A) ROC with All genes, (B) ROC10*n, (C) ROC with 7mer + Conservation, (D) ROC with TargetScan dataset, (E) ROC with miRanda dataset, and (F) ROC with PicTar dataset.

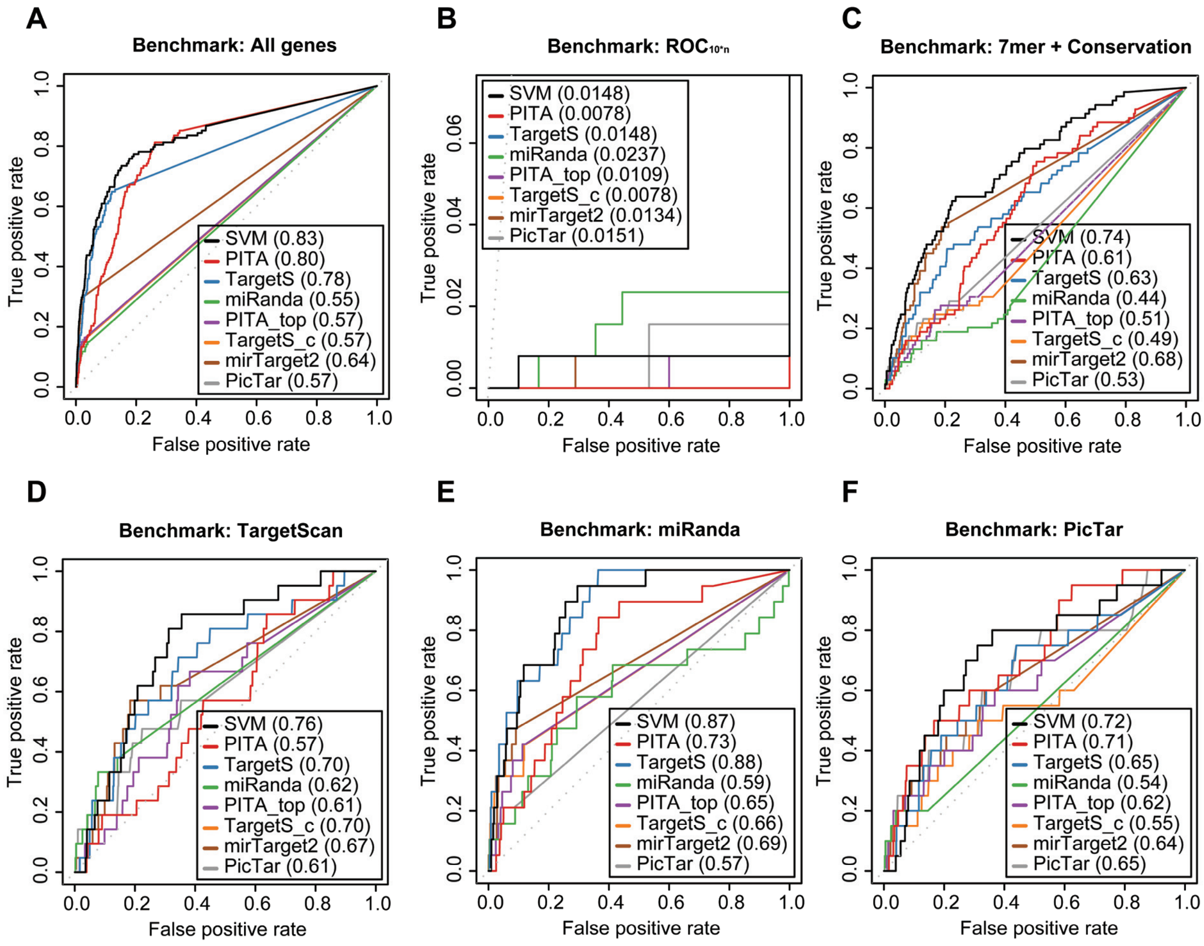

Figure S18. Benchmarks on the Linley dataset with positive record threshold by log ratio -0.3

Receiver operating characteristic (ROC) graphs show the performances of 8 different target prediction algorithms – SVM, PITA All (PITA), PITA Top (PITA_top), TargetScan (TargetS), TargetScan with conserved genes (TargetS_c), MicroCosm miRanda (miRanda), mirTarget2 and PicTar – on the Linsley dataset. Dotted lines illustrate random prediction. The ROC scores are shown in the legend box. Positive records (down-regulated) were selected by log ratio value -0.3 instead of the default value -0.2. The benchmarks used for the evaluation were (A) ROC with All genes, (B) ROC10*n, (C) ROC with 7mer + Conservation, (D) ROC with TargetScan dataset, (E) ROC with miRanda dataset, and (F) ROC with PicTar dataset.

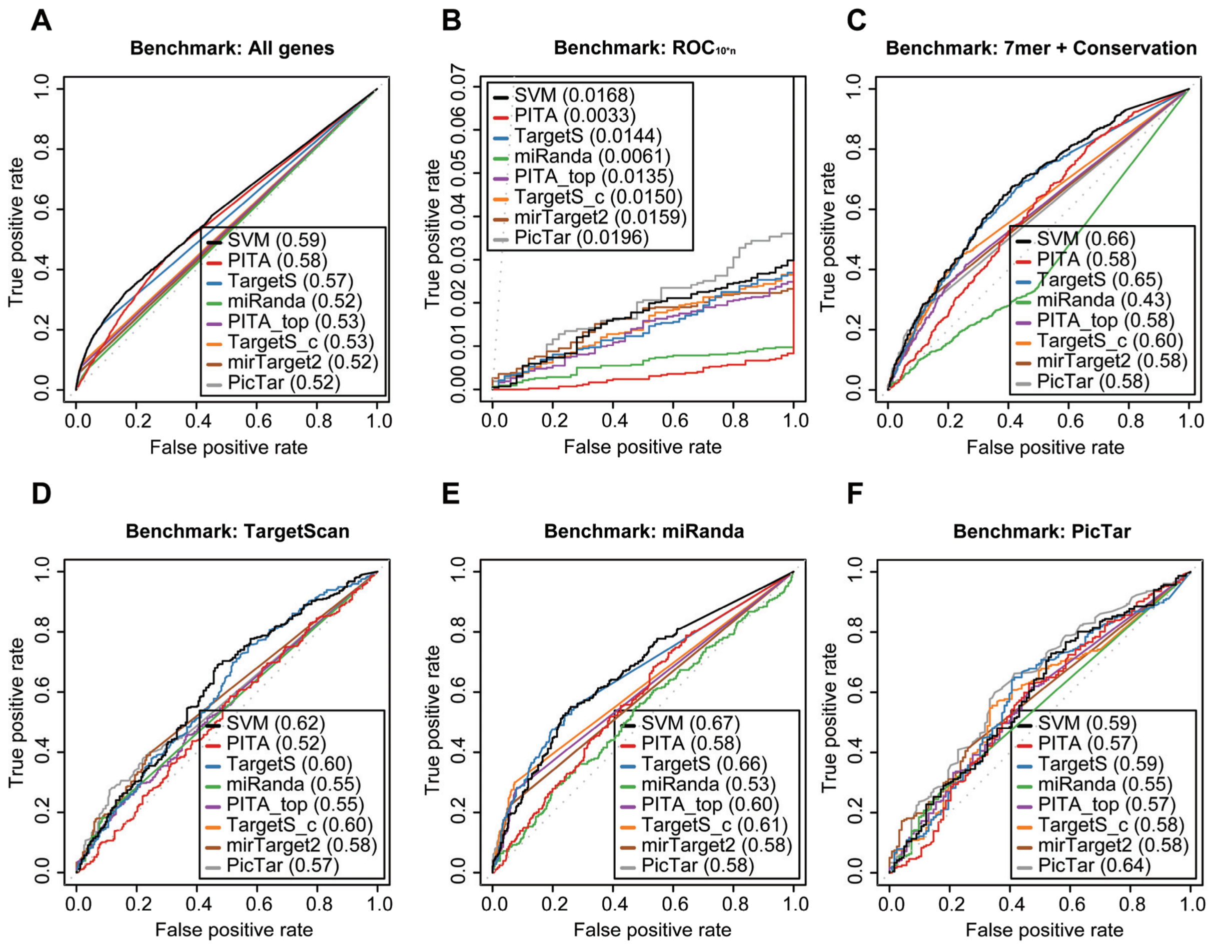

Figure S19. Benchmarks on the Selbach dataset with positive record threshold by log ratio -0.1
Receiver operating characteristic (ROC) graphs show the performances of 8 different target prediction algorithms – SVM, PITA All (PITA), PITA Top (PITA_top), TargetScan (TargetS), TargetScan with conserved genes (TargetS_c), MicroCosm miRanda (miRanda), mirTarget2 and PicTar – on the Selbach dataset. Dotted lines illustrate random prediction. The ROC scores are shown in the legend box. Positive records (down-regulated) were selected by log ratio value -0.1 instead of the default value -0.2. The benchmarks used for the evaluation were (A) ROC with All genes, (B) ROC10*n, (C) ROC with 7mer + Conservation, (D) ROC with TargetScan dataset, (E) ROC with miRanda dataset, and (F) ROC with PicTar dataset.

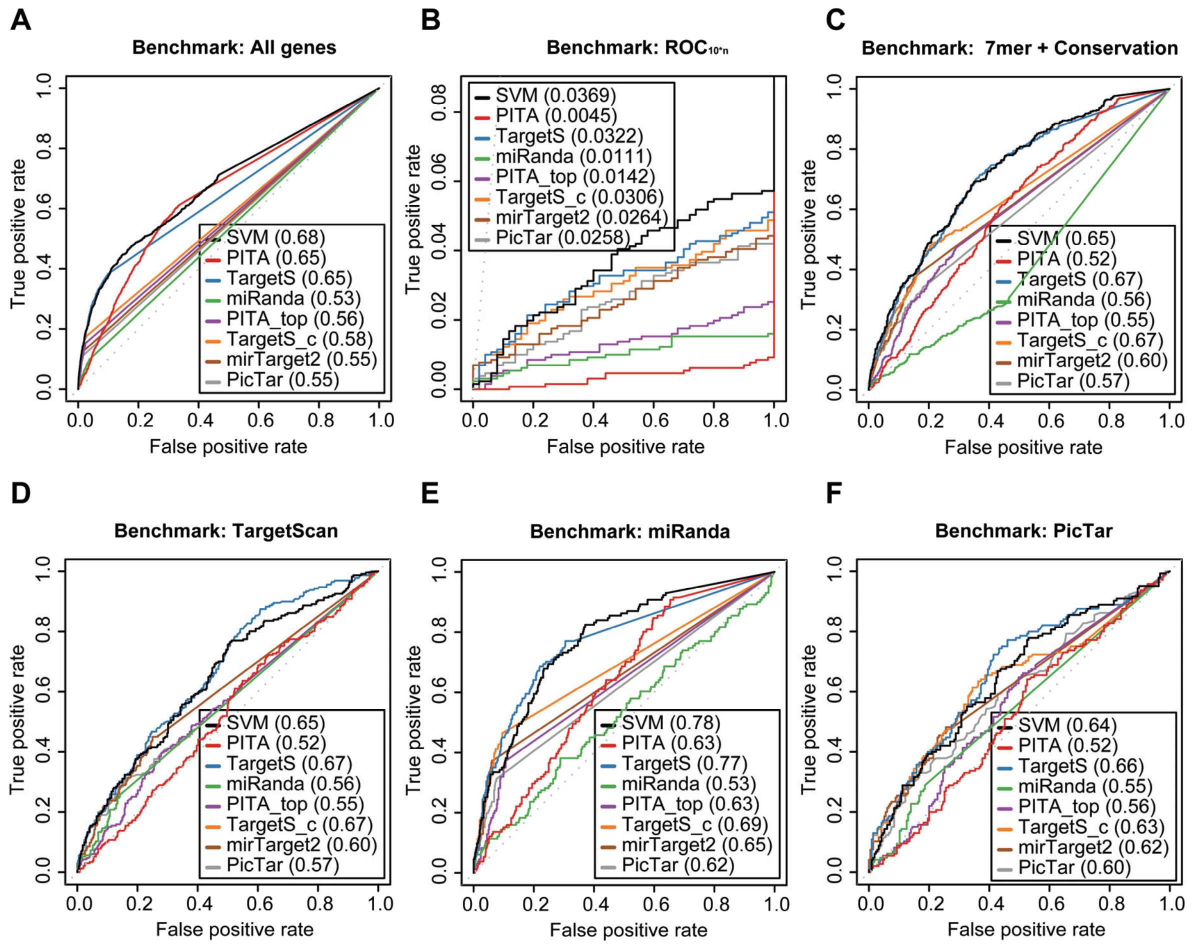

Figure S20. Benchmarks on the Selbach dataset with positive record threshold by log ratio -0.3
Receiver operating characteristic (ROC) graphs show the performances of 8 different target prediction algorithms – SVM, PITA All (PITA), PITA Top (PITA_top), TargetScan (TargetS), TargetScan with conserved genes (TargetS_c), MicroCosm miRanda (miRanda), mirTarget2 and PicTar – on the Selbach dataset. Dotted lines illustrate random prediction. The ROC scores are shown in the legend box. Positive records (down-regulated) were selected by log ratio value -0.3 instead of the default value -0.2. The benchmarks used for the evaluation were (A) ROC with All genes, (B) ROC10*n, (C) ROC with 7mer + Conservation, (D) ROC with TargetScan dataset, (E) ROC with miRanda dataset, and (F) ROC with PicTar dataset.

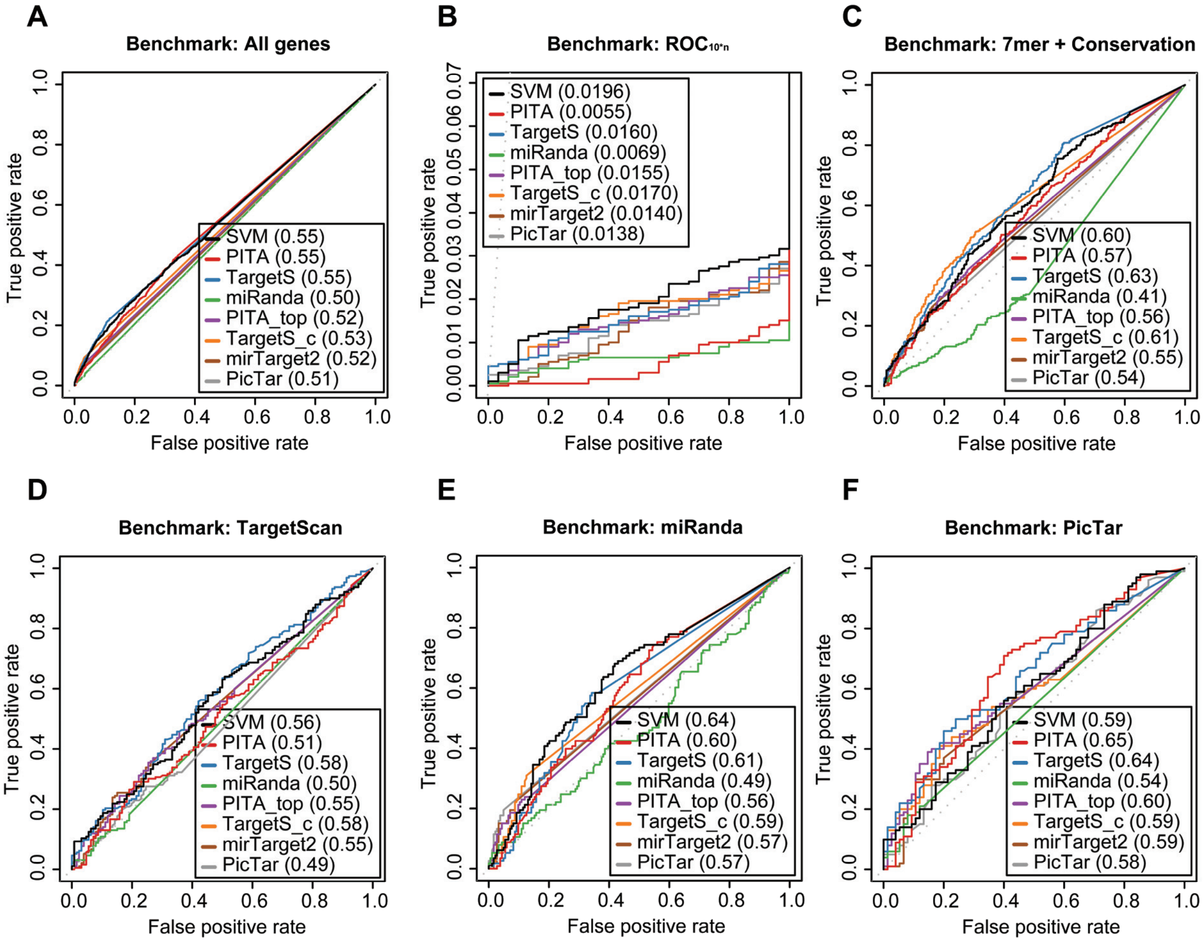

Figure S21. Benchmarks on the Baek dataset with positive record threshold by log ratio -0.1

Receiver operating characteristic (ROC) graphs show the performances of 8 different target prediction algorithms – SVM, PITA All (PITA), PITA Top (PITA_top), TargetScan (TargetS), TargetScan with conserved genes (TargetS_c), MicroCosm miRanda (miRanda), mirTarget2 and PicTar – on the Baek dataset. Dotted lines illustrate random prediction. The ROC scores are shown in the legend box. Positive records (down-regulated) were selected by log ratio value -0.1 instead of the default value -0.2. The benchmarks used for the evaluation were (A) ROC with All genes, (B) ROC10*n, (C) ROC with 7mer + Conservation, (D) ROC with TargetScan dataset, (E) ROC with miRanda dataset, and (F) ROC with PicTar dataset.

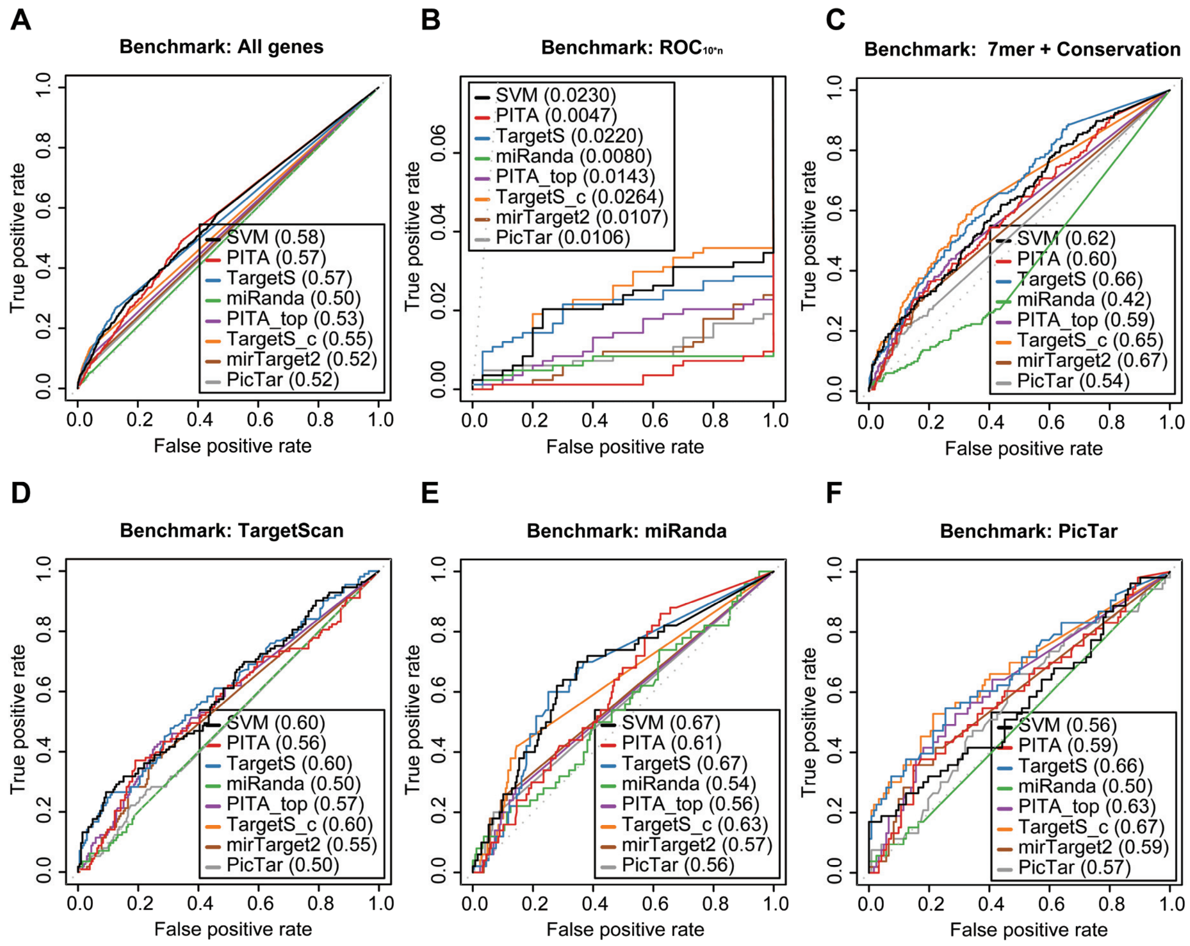

Figure S22. Benchmarks on the Baek dataset with positive record threshold by log ratio -0.3

Receiver operating characteristic (ROC) graphs show the performances of 8 different target prediction algorithms – SVM, PITA All (PITA), PITA Top (PITA_top), TargetScan (TargetS), TargetScan with conserved genes (TargetS_c), MicroCosm miRanda (miRanda), mirTarget2 and PicTar – on the Baek dataset. Dotted lines illustrate random prediction. The ROC scores are shown in the legend box. Positive records (down-regulated) were selected by log ratio value -0.3 instead of the default value -0.2. The benchmarks used for the evaluation were (A) ROC with All genes, (B) ROC10*n, (C) ROC with 7mer + Conservation, (D) ROC with TargetScan dataset, (E) ROC with miRanda dataset, and (F) ROC with PicTar dataset.

References

Baek, D., J. Villén, et al. (2008). "The impact of microRNAs on protein output." Nature.

Birmingham, A., E. M. Anderson, et al. (2006). "3'UTR seed matches,but not overall identity,are associated with RNAi off-targets." Nat Methods **3**(3): 199–204.

Enright, A. J., B. John, et al. (2003). "MicroRNA targets in Drosophila." Genome Biol **5**(1): R1.

Gaidatzis, D., E. van Nimwegen, et al. (2007). "Inference of miRNA targets using evolutionary conservation and pathway analysis." BMC Bioinformatics **8**: 69.

Gribskov, M. and N. L. Robinson (1996). "Use of receiver operating characteristic (ROC) analysis to evaluate sequence matching." Comput Chem **20**(1): 25-33.

Grimson, A., K. K. Farh, et al. (2007). "MicroRNA targeting specificity in mammals: determinants beyond seed pairing." Mol Cell **27**(1): 91-105.

Hammell, M., D. Long, et al. (2008). "mirWIP: microRNA target prediction based on microRNA-containing ribonucleoprotein-enriched transcripts." Nat Methods **5**(9): 813-819.

Huang, J. C., T. Babak, et al. (2007). "Using expression profiling data to identify human microRNA targets." Nat Methods **4**(12): 1045-1049.

Jackson, A. L., J. Burchard, et al. (2006). "Widespread siRNA "off-target" transcript silencing mediated by seed region sequence complementarity." RNA **12**(7): 1179--1187.

Kertesz, M., N. Iovino, et al. (2007). "The role of site accessibility in microRNA target recognition." Nat Genet **39**(10): 1278-1284.

Lim, L. P., N. C. Lau, et al. (2005). "Microarray analysis shows that some microRNAs downregulate large numbers of target mRNAs." Nature **433**(7027): 769--773.

Linsley, P. S., J. Schelter, et al. (2007). "Transcripts targeted by the microRNA-16 family cooperatively regulate cell cycle progression." Mol Cell Biol **27**(6): 2240-2252.

Long, D., R. Lee, et al. (2007). "Potent effect of target structure on microRNA function." Nat Struct Mol Biol **14**(4): 287-294.

Sales, G., A. Coppe, et al. (2010). "Impact of probe annotation on the integration of miRNA-mRNA expression profiles for miRNA target detection." Nucleic Acids Res.

Sethupathy, P., M. Megraw, et al. (2006). "A guide through present computational approaches for the identification of mammalian microRNA targets." Nat Methods **3**(11): 881-886.
